# Variation in heat shock protein 40 kDa relates to divergence in thermotolerance among cryptic rotifer species

**DOI:** 10.1038/s41598-022-27137-3

**Published:** 2022-12-31

**Authors:** K. Kiemel, M. Gurke, S. Paraskevopoulou, K. Havenstein, G. Weithoff, R. Tiedemann

**Affiliations:** 1 Unit of Evolutionary Biology/Systematic Zoology, Institute for Biochemistry and Biology, University of Potsdam, Karl-Liebknecht Straße 24-25, 14476 Potsdam, Germany; 2 Museum für Naturkunde – Leibniz Institute for Evolution and Biodiversity Science, Invalidenstraße 43, 10115 Berlin, Germany; 3 Department of Biology, Humboldt-University, Invalidenstraße 42, 10115 Berlin, Germany; 4 Department of Biology, Lund University, Microbiology Group, Sölvegatan 35, 223 62 Lund, Sweden; 5 Unit of Ecology and Ecosystem Modelling, Institute for Biochemistry and Biology, University of Potsdam, Am Neuen Palais 10, 14469 Potsdam, Germany

**Keywords:** Ecological genetics, Evolutionary genetics

## Abstract

Genetic divergence and the frequency of hybridization are central for defining species delimitations, especially among cryptic species where morphological differences are merely absent. Rotifers are known for their high cryptic diversity and therefore are ideal model organisms to investigate such patterns. Here, we used the recently resolved *Brachionus calyciflorus* species complex to investigate whether previously observed between species differences in thermotolerance and gene expression are also reflected in their genomic footprint. We identified a Heat Shock Protein gene (HSP 40 kDa) which exhibits cross species pronounced sequence variation. This gene exhibits species-specific fixed sites, alleles, and sites putatively under positive selection. These sites are located in protein binding regions involved in chaperoning and may therefore reflect adaptive diversification. By comparing three genetic markers (ITS, COI, HSP 40 kDa), we revealed hybridization events between the cryptic species. The low frequency of introgressive haplotypes/alleles suggest a tight, but not fully impermeable boundary between the cryptic species.

## Introduction

According to the biological species concept^1^, a species is defined as a group that is reproductively isolated from other species. Reproductive isolation can be maintained either by pre-zygotic isolation, post-zygotic isolation, or a combination of both^2^. Pre-zygotic isolation occurs before a zygote is formed and involves physiological or systemic barriers that prevent successful mating, such as differences in mating behaviour, habitat preferences or isolation through ecological or geographical barriers^3^. Post-zygotic isolation mechanisms, such as increased zygote mortality or hybrid sterility, occur after zygote formation^3^. Reproductive isolation mechanisms are however often imperfect, so that many closely related species such as cryptic species hybridize^4–7^, which counteracts species divergence or may even erode species boundaries^8^.

Cryptic species are species that require genetic markers to inform species delimitations^9–11^. Such genetically identified species not only call for a revised taxonomy^12–14^, but also pose a major challenge to evolutionary and ecological theories. Due to the lack of morphological and physiological differences, high similarity in ecological traits and adaptations is expected^15^. When these species occur in sympatry^16–19^, the principle of competitive exclusion^20^ is compromised, which further challenges the understanding of co-existence and the niche concept.

Rotifers, with their approximately 2000 described species, belong to a very diverse phylum^21^, in which an increasing number of nominal species have been declared cryptic species complexes in the last decades^22^. Among them, the monogonont freshwater *Brachionus calyciflorus* species complex has recently been subdivided into four species using genetics and morphometrics: *Brachionus calyciflorus* sensu stricto (s.s.), *Brachionus dorcas*, *Brachionus fernandoi,* and *Brachionus elevatus*^5,23^. Prior to its delimitation into four species, the *Brachionus calyciflorus* species complex had been studied with regard to molecular phylogeny^23–25^, co-existence^26^, life history characteristics^27^, phylogeography^28,29^, and reproductive isolation^28–30^. More recent studies, which have taken into account the new species classification, focus on ecological processes, e.g., niche differentiation^31^, life history traits^32^, adaptation and the underlying regulatory mechanisms^33–35^, as well as on the robustness of species boundaries through hybridisation experiments^6,7^. These studies are of great importance to understand not only how these species have diverged, but also which of their adaptations are critical for the co-existence or exclusion of certain species in a specific environment.

A previous lifetable experiment^36^ has demonstrated diverging temperature optima between *Brachionus calyciflorus* s.s. (heat-tolerant) and *Brachionus fernandoi* (heat-sensitive). Comparison of transcriptome data from the heat-tolerant *B. calyciflorus* s.s. and the heat-sensitive *B. fernandoi* revealed differential expression of Heat Shock Protein (HSP) genes relative to temperature^35^. HSP gene expression levels correlated with life history traits such that the upregulation of HSP gene expression consistently occurred when the population growth rate was low. The two species were found to exhibit optimal growth at a different temperature (*B. calyciflorus* s.s. at 26 °C and *B. fernandoi* at 20/23 °C) and increased their HSP expression outside their optimal temperature range, demonstrating a stress response of the organisms when optimal growth was not feasible^35^. The pronounced fitness differences at different thermal conditions indicate underlying mechanisms of phenotypes’ responses to environmental conditions that allow them to occur in sympatry, but in different seasons of the year^31,35^.

Temperature as an environmental variable is one of the most important drivers of evolution and diversification^37^. Temperature adaptation is particularly interesting in aquatic invertebrates which as ectotherms are stronger influenced by temperature changes than homeotherms^38^. Increased temperature accelerates ontogeny, shortens the time to maturation and the generation time^35,39–41^. The associated faster population growth can foster competitive superiority and accelerate the underlying divergent selection that enforces niche partitioning^42–44^. An organism’s adaptation to a particular environment or resource availability may occur at the protein level through the regulation of gene expression^45–48^. This gene expression regulative mechanism can be observed in situations of rapid environmental changes (i.e., length of growing seasons) in *B. plicatilis* s.s.^49,50^, or in stress response via HSP upregulation^35,51^, which is a rapid and often the first response mechanism to environmental perturbation. However, a differential regulatory response among genotypes/species to the same environmental cue (genotype x environment (G x E) variation) has to be encoded somewhere in the genome, typically upstream in gene regulatory elements (e.g., transcription factors, promoters^52,53^). In addition, a divergent adaptation to different environmental conditions may also yield adaptive differences in protein structure, i.e., altered amino acid sequences. Such changes stem from non-synonymous substitutions in the underlying protein coding genes (as the HSP genes), such that signatures of selection can occur in specific genes or distributed over the whole genome^54,55^. Evolution of gene expression and protein structure do not necessarily occur independently of each other^56,57^, and we often find their respective contribution correlating with the progress of time, i.e., altered gene expression typically evolves more rapidly than altered protein structure^53^. Furthermore, discovering signs of selection in gene sequences between different species allows us to understand the genetic underpinning of the differentiation^58,59^. When put into perspective with the divergence time, the time frame of the adaptation/speciation can be unravelled^60^.

In this study, we investigated sequence/protein evolution and divergence in the HSP 40 kDa gene, which was inferred by a candidate gene selection for temperature adaptation, both by its temperature-related differential expression^35^ and by a transcriptome-wide SNP comparison among two species of the *B. calyciflorus* species complex, i.e., the heat-tolerant species *B. calyciflorus* s.s. and the heat-sensitive *B. fernandoi.* To put HSP40kDa gene evolution into perspective, this gene was further analysed in three *Brachionus* species outside of the species complex. We hypothesise, (I) that the adaptation of *B. calyciflorus* s.s. and *B. fernandoi* to different environments is driven by divergent selection on the structure of particular proteins (here, HSP 40 kDa) and (II) that the selection for different temperatures can promote niche partitioning, and hence constitute a pre-zygotic isolation mechanism.

## Results

### Candidate gene selection

**Figure 1:**
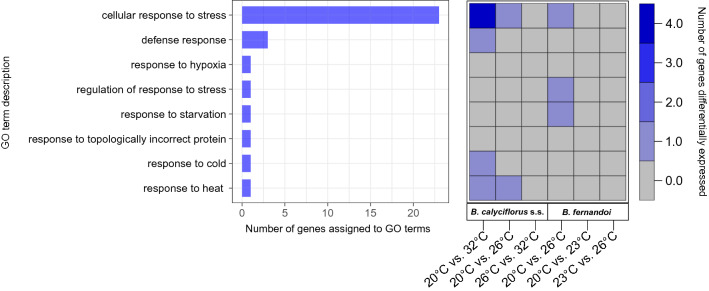
Annotation and differential expression of genes inferred to be under positive selection. Bar plot shows the genes which were assigned to the term GO:0006950 (response to stress). Bars represent the number genes assigned to one of the child terms of GO:0006950. More than one GO term can be assigned to the same gene. The heatmap shows the number of genes differentially expressed in each GO term category in *B. calyciflorus* s.s. and *B. fernandoi* under different temperatures regimes^35^.

To identify candidate genes within the *B. calyciflorus* species complex we assembled 72,165 and 94,884 super-transcripts in *B. calyciflorus* s.s and *B. fernandoi* transcriptomes, respectively. The reciprocal best blast hit identified 9655 putative orthologs which were reduced to 7976 after predicting open reading frames, locally aligning, and translating into proteins. In the selection tests, out of 7976 orthologs, 649 were significant (< 0.05) in all four pairwise Likelihood Ratio Test (LRT) comparisons, hence inferred to be putatively under positive selection. Comparing the orthologous genes under positive selection with the gene expression data^35^, 155 genes were differentially expressed relative to temperature (control: 20 °C, mild heat: *B. calyciflorus* s.s. 26 °C, *B. fernandoi* 23 °C, high heat: *B. calyciflorus* s.s. 32 °C, *B. fernandoi* 26 °C) in at least one of the two species (125 in *B. calyciflorus* s.s., 22 in *B. fernandoi,* and 8 in both species). Twenty-seven of these genes were assigned to the GO term GO:0006950 (response to stress), of which one gene was annotated to “response to heat” (GO:00009408). This gene (i.e., HSP 40 kDa) was differentially expressed in *B. calyciflorus* s.s. in cultures grown after 4 h of heat exposure to different temperature (20 °C vs. 32 °C and 20 °C vs. 26 °C), while it did not show a temperature-specific expression in *B. fernandoi* (Fig. 1).

### HSP 40 kDa sequence diversity

**Figure 2:**
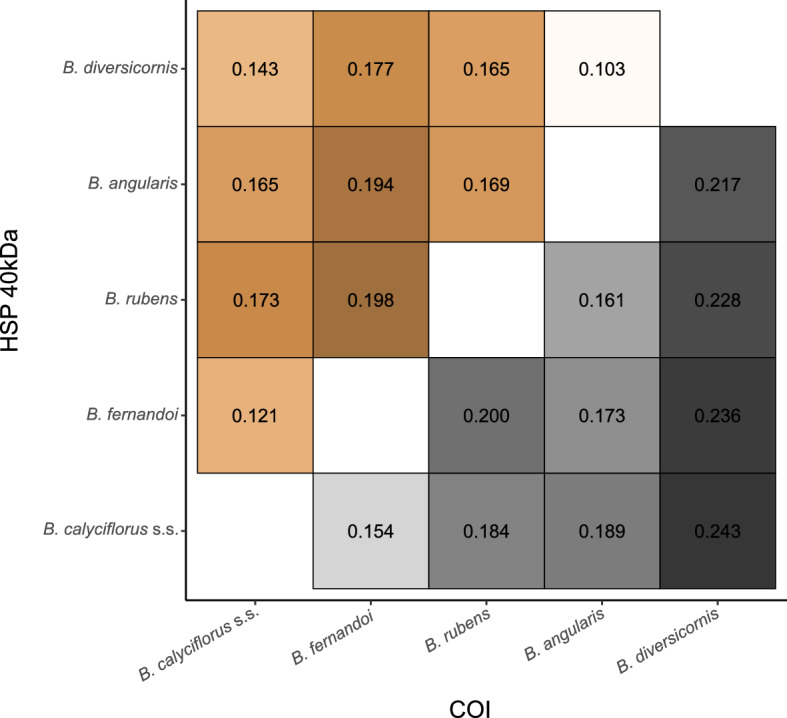
Nucleotide divergence of nuclear and mitochondrial genes. Nucleotide divergence of the nuclear HSP 40 kDa (orange) and the mitochondrial Cytochrome Oxidase Subunit I (COI) (grey) genes, based on comparisons of *B. calyciflorus* s.s., *B. fernandoi, B. rubens*, *B. angularis*, and *B. diversicornis*. The colour intensity indicates increasing nucleotide divergence between the compared sequences.

The sequence diversity of all five *Brachionus* species (*B. calyciflorus* s.s., *B. fernandoi*, *B. rubens*, *B. angularis*, and *B. diversicornis*) was assessed by comparing HSP 40 kDa and Cytochrome Oxidase Subunit I (COI) genes. We detected an overall higher nucleotide divergence in the mitochondrial COI compared to the HSP 40 kDa gene fragment (Fig. 2). For the HSP 40 kDa sequences, the lowest nucleotide divergence was detected between *B. angularis* and *B. diversicornis* (0.103), followed by *B. calyciflorus* s.s. and *B. fernandoi* (0.121). The highest nucleotide divergence was found between *B. fernandoi* and *B. rubens* (0.198). For the COI fragment, the highest nucleotide divergence was detected between *B. calyciflorus* s.s. and *B. diversicornis* (0.243), while the lowest was between *B. calyciflorus* s.s. and *B. fernandoi* (0.154). The cross-species comparison of non-synonymous (dN) vs. synonymous (dS) substitutions in the HSP 40 kDa gene yielded an overall dN/dS: 0.049, with the the highest dN/dS ratio between *B. angularis* and *B. diversicornis* (0.085), while the lowest one was found between *B. calyciflorus* s.s. and *B. diversicornis,* followed by *B. calyciflorus* s.s. and *B. fernandoi* (0.040 and 0.042, respectively) (Supplementary Fig. s4).

### Structural variation in HSP 40 kDA between *B. calyciflorus* s.s. and *B. fernandoi*

**Figure 3:**
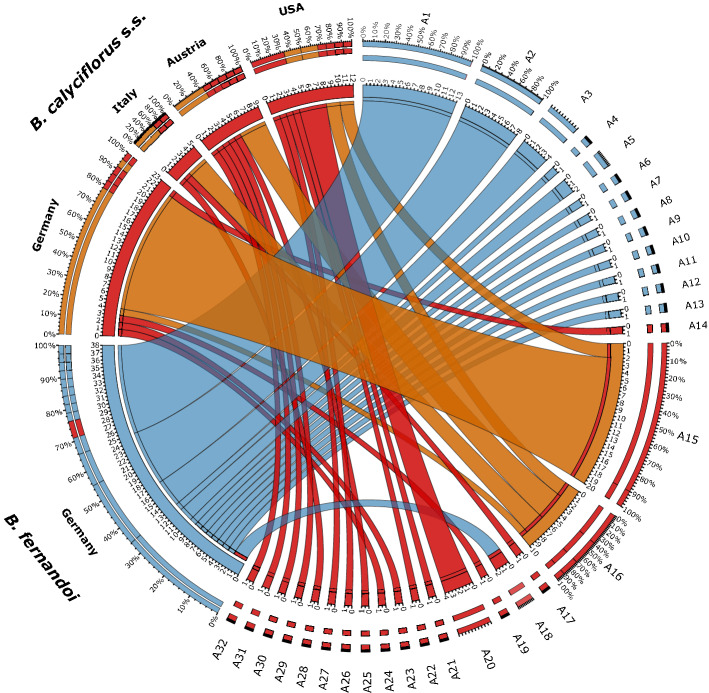
HSP 40 kDa allele identification. Circos plot of allele distribution among species *B. calyciflorus* s.s. and *B. fernandoi* and country of sample origin (Germany, Italy, Austria, USA). Labels A1-A32 stand for the identified alleles, red alleles are derived from *B. calyciflorus* s.s. and blue alleles from *B. fernandoi*. *B. calyciflorus* s.s. alleles found in more than one location are coloured orange.

**Figure 4:**
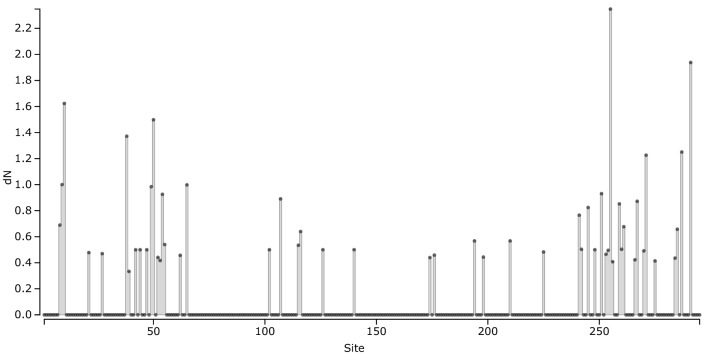
Number of non-synonymous substitutions per non-synonymous site among *B. calyciflorus* s.s. and *B. fernandoi* HSP 40 kDa for each amino acid alignment position of the 32 different HSP 40 kDa alleles.

We produced 88 genomic HSP 40kDA gene sequences for our two focus species (*B. calyciflorus* s.s., n = 50; *B. fernandoi*, n = 38) by polymerase chain reaction (PCR). The network approach based on all 88 phased sequences identified 32 unique HSP 40 kDa alleles (corresponding to 27 unique protein sequences) from which 13 alleles were exclusively found in *B. fernandoi* specimens and 19 alleles were solely found in *B. calyciflorus* s.s. specimens (Fig. 3). Only one allele, which phylogenetically clustered to the *B. calyciflorus* s.s. HSP 40 kDa allele type 18 (A18) was found in a *B. fernandoi* specimen (2484_4). The translated protein alignment (Supplementary Fig. s5) revealed 51 non-synonymous substitutions, among them the 14 found in the transcriptome data. The highest rate dN of non-synonymous substitutions per non-synonymous site among the two species was detected in position 256, followed by position 292 (Fig. 4).

### Congruence among ITS1, HSP 40 kDa, and COI species affinity

**Figure 5:**
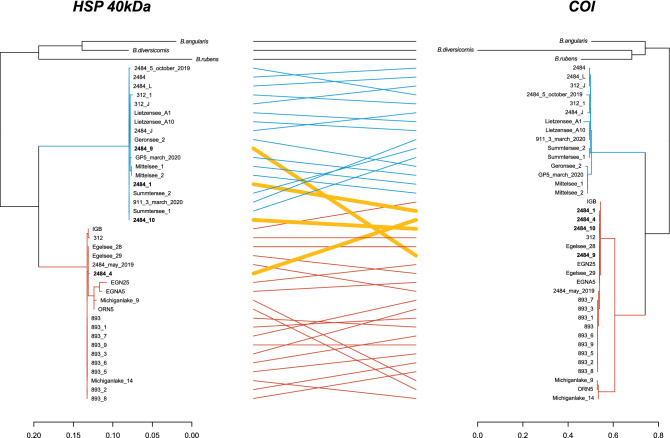
Relationship between HSP 40 kDa and COI. Tanglegram based on the nuclear HSP 40 kDa and the mitochondrial COI of species (*B. calyciflorus* s.s., *B. fernandoi, B. rubens*, *B. angularis*, *B. diversicornis*) (n = 41). Black lines indicate connections of specimens outside of the *Brachionus calyciflorus* species complex, blue lines indicate connections between specimens of *B. fernandoi* and red lines connect specimens of *B. calyciflorus* s.s. Yellow coloured connections indicate specimens originating from an introgression/hybridization event between *B. calyciflorus* s.s. and *B. fernandoi*. They all occurred at the same location and all carried a *B. fernandoi* ITS sequence*.*

The comparison of the nuclear Internal Transcribed Spacer 1 (ITS1), HSP 40 kDa, and the mitochondrial COI sequences yielded congruent species assignments for most analysed specimens. However, we identified four descendants of hybridization events between *B. calyciflorus* s.s. and *B. fernandoi* (all originating from the same pond; 2484), all of which carry the identical maternally inherited *B. calyciflorus* s.s. COI haplotype, but were assigned to *B. fernandoi* based on ITS1 sequences (Fig. 5, Supplementary Figs. s6, s7). Three of these introgressed specimens carried *B. fernandoi*—specific HSP 40 kDa alleles (2484_1, 2484_9, 2484_10), while one individual (2484_4) carried a *B. calyciflorus* s.s.—specific HSP 40 kDa allele, such that only its ITS1 sequence points towards a *B. fernandoi* affinity. Of the three introgressed specimens carrying *B. fernandoi*—specific HSP 40 kDa alleles, two were heterozygous (2484_1: A11, A1; 2484_10: A12, A2), while one was homozygous for A1 allele (2484_9). The introgressed specimen which carried a *B. calyciflorus* s.s. specific allele was homozygous for A18.

### Inferences on selection and divergence

The conducted four LRT comparisons (M0 vs. M3, M1a vs. M2a, M7 vs. M8, M8a vs. M8) of the site substitution model in PAML significantly inferred positive selection in the HSP 40 kDa gene among the five different *Brachionus* species (Table 1). The Bayes Empirical Bayes (BEB) analysis detected six sites under selection (Table 1). Visual inspection of these positively selected sites in the protein alignment (Supplementary Fig. s5) showed a species-specific protein variant pattern at one of these sites (292), while variants were shared at the other sites, but differed in their relative frequencies among the species. The comparison of the positively selected sites with the functional domain description of the HSP 40kDa^61^ revealed that four out of the six sites are located in functional domains (J-domain and C-terminal domain; Supplementary Fig. s5). The branch site model, which was conducted to identify selection and sites under selection in specific species was able to identify selection on the CD (*B. calyciflorus* s.s. & *B. fernandoi*) and D branch (*B. fernandoi*), but did not detect any species-specific sites under selection (BEBs) (Table 2). The selection scenario investigation by comparing only *B. calyciflorus* s.s. and *B. fernandoi* using a McDonald-Kreitman Test (MKT), calculated a significant Neutrality Index of 12.06, p-value: ≤ 0.001 (i.e., indicative for balancing selection or slightly deleterious mutations), while Tajima’s D (1.538) was not significant (p-value: ≥ 0.10).

**Table 1:** Likelihood ratio tests of seven different site models performed on 296 amino acids (888 bp) of the HSP 40 kDa, including 29 of the 32 unique alleles (A11, A12 and A18 were excluded from the analysis, as they only occurred in specimens affected by hybridization/introgression).

Site model	LRT	2*∆l*	df	p-value	*l*	*ω*	BEBs
M0 (one-ratio)	M0 vs. M3	262.398852	4	<< 0.001	− 3598.034542	0.0718	–
M3 (discrete)					− 3466.835116	0.1439	
M1a (nearly Neutral)	M1a vs. M2a	11.558380	2	0.003	− 3480.993265	0.1328	–
M2a (positive selection)					− 3475.214075	0.1815	
M7 (β)	M7 vs. M8	21.480488	2	<< 0.001	− 3477.904393	0.1108	–
M8 (β & ω)					− 3467.164149	0.1450	
M8a (β & ω = 1)	M8a vs. M8	31.89007	1	<< 0.001	− 3483.109184	0.0769	10*; 38*; 249**; 256*; 286*; 292**

Underlying phylogeny is based on the ITS1 (532 bp) of *B. calyciflorus* s.s., *B. fernandoi*, *B. angularis*, *B. diversicornis* and *B. rubens*. For each likelihood ratio test (LRT), we provide log likelihood (*l*) values of the compared tests (2∆*l*), degrees of freedom (df), p-values, estimated average ratio of non-synonymous vs. synonymous substitutions (ω), and the sites inferred to be under positive selection by Bayes Empirical Bayes (BEB) analysis, labeled with an * for a posterior probability of > 95% and ** for > 99%.

**Table 2:** Likelihood ratio test of branch site model performed on 296 amino acids (888 bp) of the HSP 40 kDa including the 29 of the 32 unique alleles (A11, A12 and A18 hybrid alleles are excluded from the analysis) of *B. calyciflorus* s.s. and *B. fernandoi*, *B. angularis*, *B. diversicornis* and *B. rubens*.

Branch site model	Branch	2*∆l*	df	p-value	*l*	BEBs
Selection	CD	7.398576	1	0.0065	− 3477.294043	–
Neutral					− 3480.993331	
Selection	C	0	1	1.0000	− 3480.993266	–
Neutral					− 3480.993266	
Selection	D	3.859588	1	0.0495	− 3477.544980	–
Neutral					− 3479.474774	

Phylogeny is based on 532 bp of the ITS1. For each branch selection test, we provide log likelihood (*l*) value of the compared tests (2∆*l*), degrees of freedom (df), and the p-value. No sites were inferred to be under positive selection by BEB in this test framework.

The comparison of the fixed species-specific sites revealed 11 fixed sites between *B. calyciflorus* s.s. and *B. fernandoi* (Table 3). Out of these 11 fixed sites, 9 amino acid substitutions found in *B. fernandoi* are considered benign in our Polyphen2 analysis, while two (site 63 and 66) may change protein function (inferred "possibly damaging", Table 3). Additionally, among the six positively selected sites, two amino acid substitutions may influence protein function (inferred "possibly damaging", Table 3). The inference of character polarity (ancestral vs. derived) showed a prevalence of *B. calyciflorus* s.s. to retain the ancestral variant (10/17). For position 292, the status ancestral or derived could not be identified with certainty (Table 3).

**Table 3:** Prediction of impact of non-synonymous (fixed or under selection cf. Table 1) amino acid substitutions between *B. calyciflorus* s.s. and *B. fernandoi* in comparison to *B. rubens*, *B. angularis* and *B. diversicornis*.

Site (aa)	Code	*Bc–Bf–Br–Ba–Bd*	Ancestral	Score	Sensitivity	Specificity	Prediction
10*	H10**V**/I	H–V/I–H–R–-R	H	0.002	0.99	0.30	Benign
	H10V/**I**	H–V/I–H–R–R	H	0.004	0.97	0.59	Benign
27	E27G	E–G–E–E–E	E	0.000	1.00	0.00	Benign
30	F30-	F–F–F–F	F	–	–	–	–
31	S31-	S–S–S–S	S	–	–	–	–
38*	**E**/K38E/**K**	E/K–E/K–E–E–E	E	0.231	0.91	0.88	Benign
63	L63M	L–M–L–M–L	L	0.953	0.97	0.93	Possibly damaging
66	**T**66S/F	T–F–T–F–T	T	0.143	0.92	0.86	Benign
	T66**S**/F	T–F–T–F–T	T	0.997	0.41	0.98	Possibly damaging
127	T127A	T–A–T–T–T	T	0.019	0.95	0.80	Benign
175	F175V	F–V–V–F–V	V	0.001	0.99	0.15	Benign
177	Y177N	Y–N–Y–Y–Y	Y	0.000	1.00	0.00	Benign
226	R226K	R–K–R–K–R	R	0.000	1.00	0.00	Benign
242	H242L	H–L–I–H–H	H	0.000	1.00	0.00	Benign
249*	P/**S**249P	P/S–P–P–Q–Q	E	0.116	0.93	0.86	Benign
256*	L/**F**/N256L	L/F/N–L–L–L–L	L	0.024	0.95	0.81	Benign
	L/F/**N**256L	L/F/N–L–L–L–L	L	0.094	0.93	0.85	Benign
285	D285E	D–E–D–E–D	E	0.000	1.00	0.00	Benign
286*	I/K286I	I/K–I–I–I–I	I	0.914	0.81	0.94	Possibly damaging
292*	**T**/I292R	T/I–R–A–S–V	?	0.001	0.99	0.15	Possibly damaging
	T/**I**292R	T/I–R–A–S–V	?	0.000	1.00	0.00	Benign

Predictions were made using Polyphen2. The code provides details on the amino acid substitution and the position. *Bc*–*Bf*–*Br*–*Ba*–*Bd* provides the information of the respective amino acid of *B. calyciflorus* s.s. (*Bc*), *B. fernandoi* (*Bf*), *B. rubens* (*Br*), *B. angularis* (*Ba*) and *B. diversicornis* (*Bd*). Ancestral amino acid was inferred using the most parsimonious (i.e., fewest substitutions) scenario, taking the species' phylogeny into account. In case of multiple amino acids (aa) per species, compared aa are bold in the row Code. Asteriks indicate sites under selection inferred by codeML. Note that Polyphen2 does not consider the possibility of a positive effect of an aa substitution, such that it ranks any predicted change in protein function as "damaging".

We inferred a divergence time of 25–29 Million years (Myr) between *B. calyciflorus* s.s. and *B. fernandoi*, based on mitochondrial cds, while the split between the freshwater and the marine species of *Brachionus* was estimated to be in a range of 41–47 Myr (Supplementary Fig. s8).

## Discussion

### Candidate gene selection pipeline

The utilization of available transcriptomes and different expression patterns of *B. calyciflorus* s.s. and *B. fernandoi* allowed us to identify a candidate gene which likely plays a role in the differences in temperature adaptation among the two *Brachionus* species. Indeed, HSPs have been shown to be involved in the response to various environmental stressors such as thermal stress, toxins, oxidative conditions^62–64^ and aging^61^. The importance of HSPs in the process of thermotolerance for invertebrates, particularly HSP 40 kDa, HSP 60 kDa and HSP 70 kDa, was previously shown^51^ in the monogonont rotifer *B. manjavacas.*

### Genetic variation in the HSP 40 kDa gene among *Brachionus* species

The analysis of the successfully amplified HSP 40 kDa gene of the 44 specimens of the *B. calyciflorus* cryptic species complex (*B. calyciflorus* s.s. and *B. fernandoi*) from 14 different locations (Europe, North America) revealed 32 unique alleles, indicating divergent evolution in the HSP 40 kDa. All but one allele, which originated from descendants of a hybrid, were species-specific. The high number of alleles and expressed structural differences (27 unique protein sequences) within and between the two species might be related to their cyclical parthenogenesis^65^. The dominant asexual mode of reproduction via mitotic eggs from amictic females (i.e., clones) and a less frequent switch to sexual reproduction together with a short generation time^66^ strongly reduces genomic recombination, making purifying selection to eliminate slightly deleterious mutations less effective for those organisms, a phenomenon known as Muller’s ratchet^67–69^.

The comparison between all five analysed species revealed that the species from the *B. calyciflorus* species complex are more similar (based on nucleotide divergence of the HSP 40 kDa and COI species-wise consensus sequences) to one another than to representatives from outside that species complex (*B. rubens*, *B. angularis* and *B. diversicornis*), with an overall higher nucleotide diversity in the COI. The higher diversity in the mitochondrial COI compared to nuclear HSP 40 kDa gene was expected as mitochondrial DNA in general exhibits a higher mutation rate than nuclear genes^70^, a phenomenon observed as well in rotifers^71^. While HSP 40 kDa gene divergence among *Brachionus* species generally resembled their phylogenetic affinity, the dN/dS ratio is the smallest, when comparing the not directly related species *B. diversicornis* and *B. calyciflorus* s.s. This may have arisen from similar environmental parameters that lead to similar selection pressures^72^. Indeed, a recent study found the occurrence of *B. diversicornis* to be correlated with warmer water temperatures^73^, which could indicate a higher thermotolerance—as observed in *B. calyciflorus* s.s.^35,36^—also in this species.

### Selection scenario of the HSP 40 kDa

Within the HSP 40KDa gene, six amino acid sites were inferred to be under positive selection. A closer inspection of those sites revealed that four of them are in functional domains of the gene. One site is located in the J-domain, a region known to be important in the chaperone activity of HSP 40 kDa and the HSP 70 kDa by interacting with the ATP domain of the HSP 70kDa^74^, which accelerates ATP hydrolysis^75,76^ and thus, regulates the ATPase activity^77^. Three other sites are located in the C-terminal domain of the HSP 40 kDa. Although the exact function of these sites is still undetermined, it is known that the C-terminal domain of yeast *Saccharomyces cerevisiae* contains a peptide binding site^78,79^ and is therefore enabled to interact with other proteins. Changes in amino acid composition at these sites can affect folding patterns and thus binding with other proteins (e.g., HSP 70 kDa) through suboptimal structures or polarities^80,81^. These suboptimal structures can affect the strength of HSP 70 kDa ATPase regulation and potentially alter an organism's downstream stress response, which is underlined by the predicted impact of amino acid substitutions on protein function at two of the six sites. However, among the inferred sites under positive selection, only one (292, cf. Supplementary Fig. s4) exhibited species-specific amino acids. One explanation could be that the large geographic range of our study correlated with different local selection pressures, such that most substitutions were not fixed among the entire distribution range of a species. Another explanation could be a low genomic recombination rate (Muller’s ratchet), whereby clonal organisms easily accumulate slightly deleterious mutations that can generate noise in the selection analysis, hindering the identification of species-specific sites under selection^82^. The latter is in line with the findings of the McDonald-Kreitman Test (MKT) where a high Neutrality Index of 12.05 was found between the unique alleles of *B. calyciflorus* s.s. and *B. fernandoi*. This indicates an overabundance of replacement-site polymorphisms, which can arise from slightly deleterious polymorphic variants not eliminated by purifying selection^83^, a phenomenon known from clonal organisms^84^.

Because of the potentially compromised power of selection analyses in clonal organisms^82^, we inspected all further amino acid substitutions as well. Indeed, among them eleven fixed positions between our species were identified, two of which (positions 63 and 66; cf. Table 3) with a potential impact on protein function. These sites are not located in known functional domains of the HSP 40 kDa, yet they may have an impact on the structure of the protein. The results of the branch site model test indicated positive (directional) selection in *B. fernandoi*, but not in *B. calyciflorus* s.s. This is corroborated by character polarity, i.e., *B. fernandoi* predominantly exhibiting derived substitutions, while *B. calyciflorus* s.s. mostly retaining the ancestral amino acids. This allows us to draw the conclusion that *B. fernandoi* diverged from *B. calyciflorus* s.s. A divergent selection scenario for both species is compatible with the positive Tajima’s D statistics (albeit not significant).

The substantial nucleotide divergence among the two formerly cryptic species *B. fernandoi* and *B. calyciflorus* s.s. suggest these lineages to be evolutionary more ancient than one might expect for morphologically hardly distinguishable sister species. We had inferred their divergence time as ~ 25–29 Myr. The same analysis dated the split between the *Brachionus* freshwater clade comprising *B. angularis*, *B. fernandoi* and *B. calyciflorus* s.s. and the marine clade comprising *B. plicatilis*, *B. manjavacas*, *B. rotundiformis* and *B. koreanus* to about 41–47 Myr. This latter estimate is considerably larger than an earlier estimate of divergence time between the freshwater species *B. calyciflorus* (species undefined) and the marine *B. plicatilis,* using COI and 18S as markers (~ 25 Myr^85^). This discrepancy could be due to the increased number of *Brachionus* species used in our analysis and the different genes and substitution rates used in the present study, relative to Tang et al. (2014)^85^. Tang et al. used a substitution rate of 1.76% Myr^−1^ for COI and 0.02% Myr^−1^ for 18S^85^, while we used the standard mitochondrial substitution rate for invertebrates (1.15% Myr^−1^)^86^. However, all these rates are derived from distant taxa and—to the best of our knowledge—no rotifer-specific substitution rates are available so far. Hence, these analyses should be repeated when substitution rate estimates for our target species (*B. calyciflorus* s.s. and *B. fernandoi*) become available. In any case, we note that estimated divergence times within the freshwater *B. calyciflorus* species complex are in the same order of magnitude as in the marine *B. plicatilis* species complex (represented by *B. plicatilis* and *B. manjavacas* in our analysis; cf. Supplementary Fig. s8).

### Hybridization event

Our study revealed a natural hybridization event between *B. fernandoi* and *B. calyciflorus* s.s. individuals originating at one sampling location (a single pond): Among the specimens collected there, four exhibited *B. fernandoi* ITS1 alleles, but shared the same *B. calyciflorus* s.s COI haplotype.). This comprises—to the best of our knowledge—the first reported in-situ hybridization among these two species. The ability of the species within the *B. calyciflorus* species complex to hybridize has already been suggested by the widespread occurrence of mitonuclear discordance^23^. However, only recent studies under laboratory conditions observed hybridization between the most closely related species *B. calyciflorus* s.s. and *B. elevatus*^6,7^, but with a significantly lower intraspecific fertilization (i.e., pre-zygotic isolation) and higher dormant propagule mortality (i.e., post-zygotic isolation)^6^. Another study detected signs of hybridization between *B. elevatus* and *B. dorcas*^87^. The comparison of alleles/haplotypes (HSP, ITS1, COI) in our four hybrid specimens with other samples from the same location revealed the hybrids to be F_2_ or a subsequent generation and that genes from *B. calyciflorus* s.s. introgressed into the local *B. fernandoi* gene pool. This is because all the introgressed specimens we detected in this study were homozygous for *B. fernandoi* alleles at the ITS1 locus. Furthermore, three detected hybrids carried *B. fernandoi* specific HSP 40 kDa alleles, while in one case a *B. calyciflorus* s.s. specific HSP 40 kDa allele occurred. This different genetic makeup among the hybrids suggests that they either have emerged from independent hybridization events or that the hybridization was sufficiently ancient to allow for repeated sexual recombination after the hybridization event. In any case, our findings demonstrate that *B. calyciflorus* s.s. and *B. fernandoi* can naturally hybridize at sites where they occur in sympatry. It was found in the marine *B. plicatilis* species complex that niche differentiation of very similar species (e.g., body size, biotic niches, competition abilities) is facilitated by their response to changing physical environments in combination with life and diapause history traits^16^. In the *B. calyciflorus* species complex, a previous study reported strongest pronounced differences in life history traits of *B. fernandoi* compared to *B. calyciflorus* s.s., *B. elevatus* and *B. dorcas*, such as prolonged egg and juvenile development times and an overall lower egg production rate and mictic ratio^32^. The adaptation to different temperature optima^34^, associated with differences in HSP 40 Da expression^35^ and protein structure (this study), may lead to seasonal isolation of the two species. This illustrates the above example that the niche of very similar species can be determined by their response to abiotic environmental conditions. The differential niches (here preferred temperature) occupied by the respective species reflect divergent adaptation. This acts as a pre-zygotic isolation mechanism by facilitating differences in timing of reproduction and population growth based on distinct environmental cues (i.e., temperature, photoperiod) and thus maintaining the species boundaries. Such pre-zygotic isolation by season has also been observed among *Daphnia* species (reviewed in^88^). This mechanism may however be imperfect under certain environmental conditions, leading to occasional hybridization, as observed in our study.

## Conclusion

We used transcriptome and expression data to infer a suitable candidate, the HSP 40 kDa gene, which may play a role in temperature adaptation in the *Brachionus calyciflorus* species complex. Species-specific alleles and fixed amino acid substitutions sites as well as positively selected sites located in functional domains of the protein were identified. The high number of detected alleles, the results of the branch site selection test, and the fixed sites indicate a divergent adaptation process with *B. calyciflorus* s.s. retaining ancestral features, from which *B. fernandoi* derived by positive directional selection. Furthermore, the study revealed descendants of an *in-situ* hybridization event between *B. calyciflorus* s.s. and *B. fernandoi.* This finding indicates that hybridization between those species is possible. We hypothesize that the temporally isolated niches they populate serve as pre-zygotic isolation mechanisms. This isolation may be imperfect under certain environmental conditions, yet hybridizations seem rare and seasonal niche separation generally prevents species boundaries from becoming blurred.

## Materials and methods

### Candidate gene selection

To identify candidate genes with a sequence divergence pattern related to differential temperature adaptation, previously published transcriptome data^35^ (GenBank accessions number SRA: SRR10426055-76) was analysed using the following customized bioinformatic pipeline (Supplementary Fig. s1): De novo transcriptome assemblies were used to generate a super transcript for each species using Trinity v. 2.11.0 gene splicer modeler^89^. This super transcript step was integrated to minimize redundancy in the data by inferring original genes and alternative splice forms^90^. A reciprocal best blast hit process was performed, using the blastn program of BLAST 2.9.0+^91^ to detect orthologous genes shared by *B. calyciflorus* s.s. and *B. fernandoi*. To determine the best blast hit, results were filtered using a combination of E-value, which is a size corrected measure of statistical significance, and the bit score, which is a measure of matches and mismatches between two sequences^91^. When E-values of the first two or more hits were the same, the best hit was chosen using the bit score. For each search, a list was created that contained one best hit per query. Orthologous gene pairs were extracted, and open reading frames were predicted using TransDecoder v 5.5.0^92^. To calculate ratios of the rates of non-synonymous and synonymous substitutions (dN/dS) from protein-coding regions, sequences were then translated into amino acid sequences using Biopython^93^ and locally aligned within the Biopython integrated package pairwise2 using a Smith-Waterman algorithm. Locally aligned coding sequences and the corresponding unaligned DNA sequences were used to create codon alignments using pal2nal^94^. Selection tests were conducted using all available seven (M0, M1a, M2, M3, M7, M8 and M8a) codeML models (Supplementary Table s1), as implemented in PAML v. 4.9^95^. These models where then compared in four (M0 vs. M3, M1a vs. M2, M7 vs. M8, M8 vs. M8a) predefined likelihood-ratio tests (LRT) to decide whether the different null models (i.e., models that do not allow for any codons ω > 1, corresponding to absence of positive selection) can be rejected^95^.

Orthologous genes that were significant (< 0.05) in all four LRT comparisons were annotated using Gene Ontology (GO) terms^96^. These terms are hierarchically ordered descriptions of genes or proteins molecular functions, biological processes, and cellular components. The GO term annotation was done on the online Server Argot2.5 and its batch processing function^97^. To use this function, the longest available amino acid sequence of each protein was used in a blast search against the Uniprot Swiss-Prot database^98^ and a hmmer search^99,100^ against the Pfam-A database^100^. In addition, we compared the genes exhibiting positive selection in the selection tests with those found to be differentially expressed relative to species and temperature (control: 20 °C; mild heat: *B. calyciflorus* s.s. 26 °C, *B. fernandoi* 23 °C; high heat: *B. calyciflorus* s.s. 32 °C, *B. fernandoi* 26 °C)^35^. Among those genes functionally related to temperature tolerance (GO:00009408; “response to heat”; GO:00009409; “response to cold”), only one orthologous gene, the HSP 40 kDa, exhibited signs of positive selection and was differentially expressed in more than one temperature category, hence chosen for in-depth analysis.

### DNA extraction

To extract DNA, single rotifer specimens were collected from laboratory cultures, washed in 96% EtOH and stored in a 1.5 mL reaction tube in10µL HPLC-H_2_0. In total, 56 rotifer individuals (*B. calyciflorus* s.s., n = 25; *B. fernandoi*, n = 19; *B. rubens*, n = 4; *B. diversicornis*, n = 3; *B. angularis*, n = 5), comprising 56 clonal cultures (WC medium at 20 °C under 16:8 light:dark photoperiod) which were established from one individual isolated from the field, were collected from different locations in Germany. For *B. calyciflorus* s.s., additional samples from USA, Italy and Austria were available (Supplementary Fig. s2, Supplementary Table s2). To prevent that the DNA from single individuals is lost during the extraction process, 3 µL carrier RNA [1 µg/µL] (QIAGEN, Germany) was added to the sample during the lysis. DNA was extracted using the NucleoSpin®Tissue extraction kit (Macherey–Nagel, Germany) following the manufacturer’s protocol for animal tissue (page 12–14).

### Primer design HSP 40 kDa

To amplify the HSP 40 kDa from all five different *Brachionus* species, primers were designed using the candidate gene sequences (*B. calyciflorus* s.s. and *B. fernandoi*) as well as the published HSP 40 kDa sequence of *B. calyciflorus* (unknown species assignment regarding current taxonomy; Genbank: KC176712.1^61^). Sequences were aligned in Geneious v. 8.1.9^101^ using ClustalW^102^ and the implemented primer3 algorithm^103^ was used to design primer pairs.

### Amplification and sequencing of the ITS1, COI and HSP 40 kDa

To determine the species and clonal diversity, both the nuclear internal transcribed spacer (ITS1) and the mitochondrial Cytochrome Oxidase Subunit I (COI) were amplified, using universal invertebrate primers^104,105^. PCR conditions, primer sequences, and used PCR chemicals mixes can be found in the Supplementary Tables s3, s4 and s5. The HSP 40 kDa gene of all five *Brachionus* species was amplified using newly designed primer pairs (Supplementary Tables s3, s6 and s7). Successfully amplified HSP 40 kDa, COI and ITS1 products were purified using an enzymatic (ExoAP) procedure and sequenced on a Sanger sequencing platform (Applied Biosystems™ 3500 Genetic Analyzer). Sequences from all specimens used in this study (Supplementary Table s2) were visually inspected using Geneious v. 8.1.9 and heterozygous positions (for ITS1 and HSP 40 kDa) were encoded with the standard IUPAC code for ambiguity^106^. To reconstruct both alleles of the diploid nuclear DNA, the PHASE algorithm version 2.1^107,108^ was used, as implemented in DNAsp v. 6^109^.

### HSP 40 kDa sequence diversity

To compare sequence divergence in the HSP 40 kDa gene among the five different species, consensus sequences per species were generated. In order to avoid any bias which may arise from uneven geographic sampling across species, we only used specimens originating from the same region “Uckermark” for this analysis: *B. calyciflorus* s.s. (n = 11), *B. fernandoi* (n = 8), *B. rubens* (n = 4), *B. diversicornis* (n = 3) and *B. angularis* (n = 5). Sequence ambiguities in the consensus sequence were coded as N’s (see Supplementary Table s8). Pairwise nucleotide diversities of the 1,025 bp long HSP 40 kDa gene sequence and the 435 bp long COI sequences between the five species were calculated. For an 888 bp fragment of the HSP 40 kDa gene (see below), non-synonymous resp. synonymous substitutions were identified using DNAsp v. 6.

### Expressed variation in the HSP 40 kDa gene between *B. calyciflorus* s.s. and *B. fernandoi*

Sequences originating from multiple individuals (*B. calyciflorus* s.s., n = 50; *B. fernandoi,* n = 38) were translated using the published translation reading frame of the HSP 40 kDa gene of *B. calyciflorus* (Genbank: KC176712.1). As we did not yield the complete sequence for all specimens, the alignment was truncated to 888 bp present in all sequences. Number of non-synonymous substitutions per non-synonymous site between the two species were compared using SLAC analysis implemented in Datamonkey^110,111^.

### HSP 40 kDa allele identification

To identify different allele types within the *B. calyciflorus* species complex, allele networks were generated from the resulting 88 phased sequences (*B. calyciflorus* s.s. n = 50 and *B. fernandoi* n = 38) using the program popART version 1.7^112^. Subsequently, a Circos plot^113^ was generated using the web interface based on allele types identified by popART, species, and specimens’ origin.

### Sequence divergence in the HSP 40 kDa gene relative to established barcoding markers

Species recognition within the *B. calyciflorus* complex relies on species-specific ITS1 sequences and clonal lineages are further characterized by their mitochondrial COI haplotype. For 41 specimens (*B. fernandoi* n = 18, *B. calyciflorus* s.s. n = 20, and consensus sequences of *B. rubens*, *B. angularis,* and *B. diversicornis*), sequences were available for both these marker genes and the HSP 40 kDa gene. For these specimens, congruence in genetic affinity across genetic markers was assessed using a tanglegram approach. The respective phylogenies were calculated using RaxML v.8^114^, performing 1000 bootstrap iterations. Dendrograms and the tanglegrams were generated in R v.4.0.5 with the package dendextend v.1.15.1^115^.

### Selection tests and divergence time

To test whether the expressed genetic variation among the the five different *Brachionus* species is under selection, a selection test was performed based on the 29 HSP 40 kDa species-specific alleles using the Program PAML v.4.9^95^. A specific focus was on the divergence of the two species *B. calyciflorus* s.s. and *B. fernandoi.* This analysis needs a phylogeny as input which was based on the 532 bp long ITS1 fragment and calculated using RaxML v.8, performing 1000 bootstrap iterations. The phylogeny was viewed in FigTree version 1.4.4.^116^ and adapted using Inkscape version 1.0.1.^117^ (Supplementary Fig. s3). This ITS1 phylogeny as well as the HSP 40 kDa unique alleles were used to infer sites under selection according to the codeML site model implemented in PAML, testing all seven (M0, M1a, M2, M3, M7, M8 and M8a) different substitution models. The different models vary regarding substitution rates of synonymous and non-synonymous sites (ω values; see Supplementary Table s1) with a subsequent site under selection inference via Bayes Empirical Bayes (BEBs) (for parameter setting see Supplementary Table s9). The models were compared in four (M0 vs. M3, M1a vs. M2, M7 vs. M8, M8a vs. M8) predefined Likelihood Ratio Tests (LRTs) to decide whether the respective null models can be rejected. Significance of the M0 vs. M3 comparison indicates variation in ω, a prerequisite for further tests on positive selection. The null model M1a allows only negatively selected and neutral sites (2 ω values), to which M2a adds a third ω for positively selected sites. M7 and M8 resemble M1a and M2a, but allow for rate variation among negatively selected sites (10 ω values < 1 taken from a β distribution), without consideration of neutral sites (ω = 1). The last comparison of M8a vs. M8 tests for positive selection against a null model (M8a) resembling M7, but with an additional fixed ω = 1 (allowing for sites under selective neutrality); this comparison has been found to increase robustness by yielding fewer false positive results^118^. An additional branch site test was performed to identify patterns of selection on three different branches: (I) the shared branch of *B. calyciflorus* s.s. and *B. fernandoi*, (II) *B. calyciflorus* s.s. only and (III) *B. fernandoi* only (Supplementary Fig. s3). Subsequently, to further compare the two formerly cryptic species of *B. calyciflorus* s.s. and *B. fernandoi*, a codon-based McDonald–Kreitman test (MKT) and Tajima’s D statistics (allele frequency based) were calculated based on the unique inferred alleles using DNAsp v. 6.

Species-specific fixed amino acid substitutions within the *B. calyciflorus* species complex were identified (Supplementary Fig. s5) and the ancestral amino acid was inferred under parsimony, using sequence information from *B. rubens*, *B. angularis* and *B. diversicornis* and the ITS1 phylogeny (Supplementary Fig. s3). The functional effects of the specific amino acid (aa) substitutions were predicted with Polyphen2^119^ using *B. calyciflorus* s.s. as a reference. Note here that Polyphen2 does not consider the possibility of a positive effect of an aa substitution, such that it ranks any substitution without a predicted change in protein function as "benign" (i.e., no effect), while any predicted change is ranked as "damaging" (i.e., predicted to impact protein function).

To estimate the within *B. calyciflorus* species complex divergence time between *B. calyciflorus* s.s. and *B. fernandoi* the program BEAST v.1.10.4^120^ was used. Whole mtGenome coding sequences (cds) of different rotifer species were downloaded from NCBI: *B. calyciflorus* s.s.^121^, *B. fernandoi*^121^, *B. angularis*^122^, *B. manjavacas*^123^, *B.* *plicatilis*^124^, *B.* *koreanus*^125^, *B.* *rotundiformis*^126^, *B.* *rubens*^127^ and *P. similis*^128^ (for Genebank accession numbers cf. Supplementary figure s8). In absence of a published substitution rate for rotifers, the standard divergence rate for invertebrates/insects (2.3% Myr^−1^) with a constant substitution rate of 0.0115 per million years per lineages^86^ was used to calibrate the phylogeny. Cds were aligned in Geneious v. 8.1.9 using ClustalW and the IQ-Tree webserver^129^ was used to determine the best fitting substitution model. The BEAST run was conducted with 10 million MCMC iterations, with GTR + G4 + I chosen as the substitution model. Convergence was checked with Tracer v.1.7.2^130^, using the first million MCMC iterations as the burn-in value and tree was visualized using FigTree version 1.4.4. and adapted in Inkscape version 1.0.1.

## Supplementary Information

Supplementary Information.

## Data Availability

The genome sequence data (ITS1, COI and HSP 40 kDa) that supports the findings of this study are openly available on Genbank of NCBI (http://www.ncbi.nlm.nih.gov/) under the Accession numbers: ITS1: OP868747-OP868802, COI: OP861581-OP861626 and HSP 40 kDa: OP888060-OP888091. The associated BioProject, SRA, and Bio-Sample numbers are PRJNA544636, SRR10426055—SRR10426076 and SAMN11845726—SAMN11845747.

## References

[1] Mayr E (1942). Systematics and the Origin of Species, from the Viewpoint of a Zoologist.

[2] Ostevik KL, Andrew RL, Otto SP, Rieseberg LH (2016). Multiple reproductive barriers separate recently diverged sunflower ecotypes. Evolution.

[3] Seehausen O (2014). Genomics and the origin of species. Nat. Rev. Genet..

[4] Cheng J, Sha Z-L (2017). Cryptic diversity in the Japanese mantis shrimp (Crustacea: Squillidae): Allopatric diversification, secondary contact and hybridization. Sci. Rep..

[5] Michaloudi E (2018). Reverse taxonomy applied to the *Brachionus calyciflorus* cryptic species complex: Morphometric analysis confirms species delimitations revealed by molecular phylogenetic analysis and allows the (re)description of four species. PLoS ONE.

[6] Zhang W, Declerck SAJ (2022). Intrinsic postzygotic barriers constrain cross-fertilisation between two hybridising sibling rotifer species of the *Brachionus calyciflorus* species complex. Freshw. Biol..

[7] Zhang W, Declerck SAJ (2022). Reduced fertilization constitutes an important prezygotic reproductive barrier between two sibling species of the hybridizing *Brachionus calyciflorus* species complex. Hydrobiologia.

[8] Seehausen O, van Alphen JJM, Witte F (1997). Cichlid fish diversity threatened by eutrophication that curbs sexual selection. Science.

[9] Bickford D (2007). Cryptic species as a window on diversity and conservation. Trends Ecol. Evol..

[10] Gill BA (2016). Cryptic species diversity reveals biogeographic support for the ’mountain passes are higher in the tropics’ hypothesis. Proc. R. Soc. B..

[11] Sáez AG, Lozano E (2005). Body doubles. Nature.

[12] Fišer C, Robinson CT, Malard F (2018). Cryptic species as a window into the paradigm shift of the species concept. Mol. Ecol..

[13] Mills S (2017). Fifteen species in one: deciphering the *Brachionus plicatilis* species complex (Rotifera, Monogononta) through DNA taxonomy. Hydrobiologia.

[14] Struck TH (2018). Finding evolutionary processes hidden in cryptic species. Trends Ecol. Evol..

[15] Leibold MA, McPeek MA (2006). Coexistence of the niche and neutral perspectives in community ecology. Ecology.

[16] Gabaldón C, Fontaneto D, Carmona MJ, Montero-Pau J, Serra M (2017). Ecological differentiation in cryptic rotifer species: What we can learn from the *Brachionus plicatilis* complex. Hydrobiologia.

[17] Nicholls B, Racey PA (2006). Contrasting home-range size and spatial partitioning in cryptic and sympatric pipistrelle bats. Behav. Ecol. Sociobiol..

[18] Ortells R, Gómez A, Serra M (2003). Coexistence of cryptic rotifer species: Ecological and genetic characterisation of Brachionus plicatilis. Freshw. Biol..

[19] Wellborn GA, Cothran RD (2007). Niche diversity in crustacean cryptic species: Complementarity in spatial distribution and predation risk. Oecologia.

[20] Gause GF (1934). The struggle for existence.

[21] Segers H (2008). Global diversity of rotifers (Rotifera) in freshwater. Hydrobiologia.

[22] Fontaneto D (2014). Molecular phylogenies as a tool to understand diversity in rotifers. Int. Rev. Hydrobiol..

[23] Papakostas S (2016). Integrative taxonomy recognizes evolutionary units despite widespread mitonuclear discordance: Evidence from a rotifer cryptic species complex. Syst. Biol..

[24] García-Morales AE, Elías-Gutiérrez M (2013). DNA barcoding of freshwater rotifera in Mexico: Evidence of cryptic speciation in common rotifers. Mol. Ecol. Resour..

[25] Wang XL (2014). Differences in life history characteristics between two sibling species in *Brachionus calyciflorus* complex from tropical shallow lakes. Ann. Limnol. Int. J. Lim..

[26] Wen X, Xi Y, Zhang G, Xue Y, Xiang X (2016). Coexistence of cryptic Brachionus calyciflorus (Rotifera) species: Roles of environmental variables. J. Plankton Res..

[27] Xiang X-L, Chen Y-Y, Han Y, Wang X-L, Xi Y-L (2016). Comparative studies on the life history characteristics of two *Brachionus calyciflorus* strains belonging to the same cryptic species. Biochem. Syst. Ecol..

[28] Xiang X-L (2011). Patterns and processes in the genetic differentiation of the *Brachionus calyciflorus* complex, a passively dispersing freshwater zooplankton. Mol. Phylogenet. Evol..

[29] Xiang X-L (2011). Genetic differentiation and phylogeographical structure of the *Brachionus calyciflorus* complex in eastern China. Mol. Ecol..

[30] Gilbert JJ, Walsh EJ (2005). *Brachionus calyciflorus* is a species complex: Mating behavior and genetic differentiation among four geographically isolated strains. Hydrobiologia.

[31] Zhang Y (2018). Temporal patterns and processes of genetic differentiation of the *Brachionus calyciflorus* (Rotifera) complex in a subtropical shallow lake. Hydrobiologia.

[32] Zhang W, Lemmen KD, Zhou L, Papakostas S, Declerck SAJ (2019). Patterns of differentiation in the life history and demography of four recently described species of the *Brachionus calyciflorus* cryptic species complex. Freshw. Biol..

[33] Lemmen KD, Verhoeven KJF, Declerck SAJ (2022). Experimental evidence of rapid heritable adaptation in the absence of initial standing genetic variation. Funct. Ecol..

[34] Paraskevopoulou S, Dennis AB, Weithoff G, Hartmann S, Tiedemann R (2019). Within species expressed genetic variability and gene expression response to different temperatures in the rotifer *Brachionus calyciflorus* sensu stricto. PLoS ONE.

[35] Paraskevopoulou S, Dennis AB, Weithoff G, Tiedemann R (2020). Temperature-dependent life history and transcriptomic responses in heat-tolerant versus heat-sensitive *Brachionus* rotifers. Sci. Rep..

[36] Paraskevopoulou S, Tiedemann R, Weithoff G (2018). Differential response to heat stress among evolutionary lineages of an aquatic invertebrate species complex. Biol. Lett..

[37] Takemoto K, Akutsu T (2008). Origin of structural difference in metabolic networks with respect to temperature. BMC Syst. Biol..

[38] Angilletta MJ (2009). Thermal Adaptation: A Theoretical and Empirical Synthesis.

[39] Atkinson D (1994). Temperature and organism size: A biological law for ectotherms?. Adv. Ecol. Res..

[40] Gillooly JF, Brown JH, West GB, Savage VM, Charnov EL (2001). Effects of size and temperature on metabolic rate. Science.

[41] Walczyńska A, Franch-Gras L, Serra M (2017). Empirical evidence for fast temperature-dependent body size evolution in rotifers. Hydrobiologia.

[42] Brown WL, Wilson EO (1956). Character displacement. Syst. Zool..

[43] Marrone F, Fontaneto D, Naselli-Flores L (2022). Cryptic diversity, niche displacement and our poor understanding of taxonomy and ecology of aquatic microorganisms. Hydrobiologia.

[44] Pekkonen M, Ketola T, Laakso JT (2013). Resource availability and competition shape the evolution of survival and growth ability in a bacterial community. PLoS ONE.

[45] Brawand D (2011). The evolution of gene expression levels in mammalian organs. Nature.

[46] Drummond DA, Wilke CO (2009). The evolutionary consequences of erroneous protein synthesis. Nat. Rev. Genet..

[47] Fraser HB (2011). Genome-wide approaches to the study of adaptive gene expression evolution: Systematic studies of evolutionary adaptations involving gene expression will allow many fundamental questions in evolutionary biology to be addressed. BioEssays.

[48] Fraser HB (2013). Gene expression drives local adaptation in humans. Genome Res..

[49] Franch-Gras L (2019). Rotifer adaptation to the unpredictability of the growing season. Hydrobiologia.

[50] Tarazona E, Lucas-Lledó JI, Carmona MJ, García-Roger EM (2020). Gene expression in diapausing rotifer eggs in response to divergent environmental predictability regimes. Sci. Rep..

[51] Smith HA, Burns AR, Shearer TL, Snell TW (2012). Three heat shock proteins are essential for rotifer thermotolerance. J. Exp. Mar. Biol. Ecol..

[52] Alonso CR, Wilkins AS (2005). The molecular elements that underlie developmental evolution. Nat. Rev. Genet..

[53] Romero IG, Ruvinsky I, Gilad Y (2012). Comparative studies of gene expression and the evolution of gene regulation. Nat. Rev. Genet..

[54] Franch-Gras L (2018). Genomic signatures of local adaptation to the degree of environmental predictability in rotifers. Sci. Rep..

[55] Nowell RW (2018). Comparative genomics of bdelloid rotifers: Insights from desiccating and nondesiccating species. PLoS Biol..

[56] Feugeas J-P (2016). Links between transcription, environmental adaptation and gene variability in *Escherichia coli*: Correlations between gene expression and gene variability reflect growth efficiencies. Mol. Biol. Evol..

[57] Pai AA, Pritchard JK, Gilad Y (2015). The genetic and mechanistic basis for variation in gene regulation. PLoS Genet..

[58] Gribble KE, Mark Welch DB (2012). The mate recognition protein gene mediates reproductive isolation and speciation in the *Brachionus plicatilis* cryptic species complex. BMC Evol. Biol..

[59] Via S (2009). Natural selection in action during speciation. Proc. Natl. Acad. Sci. USA..

[60] Ho SYW, Duchêne S (2014). Molecular-clock methods for estimating evolutionary rates and timescales. Mol. Ecol..

[61] Yang J, Mu Y, Dong S, Jiang Q, Yang J (2014). Changes in the expression of four heat shock proteins during the aging process in *Brachionus calyciflorus* (Rotifera). Cell Stress Chaperones.

[62] Mahmood K, Jadoon S, Mahmood Q, Irshad M, Hussain J (2014). Synergistic effects of toxic elements on heat shock proteins. Biomed. Res. Int..

[63] Park JC (2020). Genome-wide identification and structural analysis of heat shock protein gene families in the marine rotifer *Brachionus* spp.: Potential application in molecular ecotoxicology. Comp. Biochem. Physiol. D.

[64] Santoro M (2000). Heat shock factors and the control of the stress response. Biochem. Pharmacol..

[65] Birky CW, Gilbert JJ (1971). Parthenogenesis in rotifers: The control of sexual and asexual reproduction. Am. Zool..

[66] Snell TW (2014). Rotifers as models for the biology of aging. Int. Rev. Hydrobiol..

[67] Felsenstein J (1974). The evolutionary advantage of recombination. Genetics.

[68] Muller HJ (1932). Some genetic aspects of sex. Am. Nat..

[69] Muller HJ (1964). The relation of recombination to mutational advance. Mut. Res..

[70] Ballard JWO, Whitlock MC (2004). The incomplete natural history of mitochondria. Mol. Ecol..

[71] Zhang Y, Xu S, Sun C, Dumont H, Han B-P (2021). A new set of highly efficient primers for COI amplification in rotifers. Mitochondrial DNA B.

[72] Turner CB, Marshall CW, Cooper VS (2018). Parallel genetic adaptation across environments differing in mode of growth or resource availability. Evol. Lett..

[73] Lan B (2021). Tempo-spatial variations of zooplankton communities in relation to environmental factors and the ecological implications: A case study in the hinterland of the Three Gorges Reservoir area. China. PLoS ONE.

[74] Pellecchia M, Szyperski T, Wall D, Georgopoulos C, Wüthrich K (1996). NMR structure of the J-domain and the Gly/Phe-rich region of the *Escherichia coli* DnaJ chaperone. Mol. Biol..

[75] Greene MK, Maskos K, Landry SJ (1998). Role of the J-domain in the cooperation of Hsp40 with Hsp70. Proc. Natl. Acad. Sci. USA.

[76] Wittung-Stafshede P, Guidry J, Horne BE, Landry SJ (2003). The J-domain of Hsp40 couples ATP hydrolysis to substrate capture in Hsp70. Biochemistry.

[77] Cintron NS, Toft D (2006). Defining the requirements for Hsp40 and Hsp70 in the Hsp90 chaperone pathway. J. Biol. Chem..

[78] Li J, Qian X, Sha B (2003). The crystal structure of the yeast Hsp40 Ydj1 complexed with its peptide substrate. Structure.

[79] Sha B, Lee S, Cyr DM (2000). The crystal structure of the peptide-binding fragment from the yeast Hsp40 protein Sis1. Structure.

[80] Brender JR, Zhang Y (2015). Predicting the effect of mutations on protein-protein binding interactions through structure-based interface profiles. PLoS Comput. Biol..

[81] Shortle D (2009). One sequence plus one mutation equals two folds. Proc. Natl. Acad. Sci. USA.

[82] Charlesworth B (2012). The effects of deleterious mutations on evolution at linked sites. Genetics.

[83] Cutter AD (2019). A Primer of Molecular Population Genetics.

[84] Barraclough TG, Fontaneto D, Ricci C, Herniou EA (2007). Evidence for inefficient selection against deleterious mutations in cytochrome oxidase I of asexual bdelloid rotifers. Mol. Biol. Evol..

[85] Tang CQ, Obertegger U, Fontaneto D, Barraclough TG (2014). Sexual species are separated by larger genetic gaps than asexual species in rotifers. Evol. Int. J. Org. Evol..

[86] Brower AV (1994). Rapid morphological radiation and convergence among races of the butterfly *Heliconius erato* inferred from patterns of mitochondrial DNA evolution. Proc. Natl. Acad. Sci. U.S.A..

[87] Yang W, Deng Z, Blair D, Hu W, Yin M (2022). Phylogeography of the freshwater rotifer *Brachionus calyciflorus* species complex in China. Hydrobiologia.

[88] Chin TA, Cristescu ME (2021). Speciation in *Daphnia*. Mol. Ecol..

[89] Grabherr MG (2011). Full-length transcriptome assembly from RNA-seq data without a reference genome. Nat. Biotechnol..

[90] Davidson NM, Hawkins ADK, Oshlack A (2017). SuperTranscripts: A data driven reference for analysis and visualisation of transcriptomes. Genome Biol..

[91] Altschul SF, Gish WP, Miller W, Myers EW, Lipman DL (1990). Basic local alignment search tool. Mol. Biol..

[92] Haas BJ (2013). De novo transcript sequence reconstruction from RNA-seq using the Trinity platform for reference generation and analysis. Nat. Protoc..

[93] Cock PJA (2009). Biopython: Freely available Python tools for computational molecular biology and bioinformatics. Bioinformatics.

[94] Suyama M, Torrents D, Bork P (2006). PAL2NAL: Robust conversion of protein sequence alignments into the corresponding codon alignments. Nucleic Acids Res..

[95] Yang Z (2007). PAML 4: Phylogenetic analysis by maximum likelihood. Mol. Biol. Evol..

[96] Ashburner M (2000). Gene ontology: Tool for the unification of biology. The Gene Ontology Consortium. Nat. Genet..

[97] Lavezzo E, Falda M, Fontana P, Bianco L, Toppo S (2016). Enhancing protein function prediction with taxonomic constraints: The Argot2.5 web server. Methods.

[98] The UniProt Consortium (2021). UniProt: The universal protein knowledgebase in 2021. Nucleic Acids Res..

[99] Finn RD, Clements J, Eddy SR (2011). HMMER web server: Interactive sequence similarity searching. Nucleic Acids Res..

[100] Finn RD (2014). Pfam: the protein families database. Nucleic Acids Res..

[101] Kearse M (2012). Geneious Basic: An integrated and extendable desktop software platform for the organization and analysis of sequence data. Bioinformatics.

[102] Thompson JD, Higgins DG, Gibson TJ (1994). CLUSTAL W: Improving the sensitivity of progressive multiple sequence alignment through sequence weighting, position-specific gap penalties and weight matrix choice. Nucleic Acids Res..

[103] Untergasser A (2012). Primer3-new capabilities and interfaces. Nucleic Acids Res..

[104] Palumbi SR (1996). The polymerase chain reaction. Mol. Syst..

[105] Folmer O, Black M, Hoeh W, Lutz R, Vrijenhoek R (1994). DNA primers for amplification of mitochondrial cytochrome c oxidase subunit I from diverse metazoan invertebrates. Mol. Mar. Biol. Biotechnol..

[106] Cornish-Bowden A (1985). Nomenclature for incompletely specified bases in nucleic acid sequences: Recommendations. Nucleic Acids Res..

[107] Stephens M, Smith NJ, Donnelly P (2001). A new statistical method for haplotype reconstruction from population data. Am. J. Hum. Genet..

[108] Stephens M, Donnelly P (2003). A Comparison of bayesian methods for haplotype reconstruction from population genotype data. Am. J. Hum. Genet..

[109] Rozas J (2017). DnaSP 6: DNA sequence polymorphism analysis of large data sets. Mol. Biol. Evol..

[110] Kosakovsky Pond SL, Frost SDW (2005). Not so different after all: A comparison of methods for detecting amino acid sites under selection. Mol. Biol. Evol..

[111] Weaver S (2018). Datamonkey 2.0: A modern web application for characterizing selective and other evolutionary processes. Mol. Biol. Evol..

[112] Leigh JW, Bryant D (2015). popart: Full-feature software for haplotype network construction. Methods Ecol. Evol..

[113] Krzywinski M (2009). Circos: An information aesthetic for comparative genomics. Genome Res..

[114] Stamatakis A (2014). RAxML version 8: A tool for phylogenetic analysis and post-analysis of large phylogenies. Bioinformatics.

[115] Galili T (2015). dendextend: An R package for visualizing, adjusting and comparing trees of hierarchical clustering. Bioinformatics.

[116] Andrew Rambaut Group. *FigTree*. (2022). http://tree.bio.ed.ac.uk/software/.

[117] Inkscape Project. *Inkscape*. (2020). https://inkscape.org.

[118] Wong WSW, Yang Z, Goldman N, Nielsen R (2004). Accuracy and power of statistical methods for detecting adaptive evolution in protein coding sequences and for identifying positively selected sites. Genetics.

[119] Adzhubei IA (2010). A method and server for predicting damaging missense mutations. Nat. Methods.

[120] Suchard MA (2018). Bayesian phylogenetic and phylodynamic data integration using BEAST 1.10. Virus Evol..

[121] Kiemel K, de Cahsan B, Paraskevopoulou S, Weithoff G, Tiedemann R (2022). Mitochondrial genomes of the freshwater monogonont rotifer Brachionus fernandoi and of two additional B. calyciflorus sensu stricto lineages from Germany and the USA (Rotifera, Brachionidae). Mitochondrial DNA B.

[122] Kim M-S (2020). Complete mitochondrial genome of the freshwater monogonont rotifer *Brachionus angularis* (Rotifera, Brachionidae). Mitochondrial DNA B..

[123] Kim M-S (2021). Complete mitochondrial genomes of two marine monogonont rotifer *Brachionus manjavacas* strains. Mitochondrial DNA B..

[124] Suga K, Mark Welch DB, Tanaka Y, Sakakura Y, Hagiwara A (2008). Two circular chromosomes of unequal copy number make up the mitochondrial genome of the rotifer Brachionus plicatilis. Mol. Biol. Evol..

[125] Hwang D-S (2014). Complete mitochondrial genome of the monogonont rotifer, Brachionus koreanus (Rotifera, Brachionidae). Mitochondrial DNA B..

[126] Kim H-S (2017). Complete mitochondrial genome of the monogonont rotifer Brachionus rotundiformis (Rotifera, Brachionidae). Mitochondrial DNA B..

[127] Choi B-S (2019). Complete mitochondrial genome of the freshwater monogonont rotifer Brachionus rubens (Rotifera, Brachionidae). Mitochondrial DNA B..

[128] Choi B-S (2020). Complete mitochondrial genome of the marine monogonont rotifer Proales similis (Rotifera, Proalidae). Mitochondrial DNA B..

[129] Trifinopoulos J, Nguyen L-T, von Haeseler A, Minh BQ (2016). W-IQ-TREE: A fast online phylogenetic tool for maximum likelihood analysis. Nucleic Acids Res..

[130] Drummond AJ, Rambaut A (2007). BEAST: Bayesian evolutionary analysis by sampling trees. BMC Evol. Biol..

