# Supplementary material for: Variation in heat shock protein 40 kDa relates to divergence in thermotolerance among cryptic rotifer species
Source: Sci Rep. 2022 Dec 31;12:22626. doi: 10.1038/s41598-022-27137-3 (PMC9805463; doi:10.1038/s41598-022-27137-3)
Supplement: Supplementary file 1 — Supplementary Information. [file 41598_2022_27137_MOESM1_ESM.docx]

***Supplementary Material***

Variation in Heat Shock Protein 40kDa relates to divergence in thermotolerance among cryptic rotifer species

Kiemel K., Gurke M., Paraskevopoulou S., Havenstein K., Weithoff G., Tiedemann R.

**Table of Contents:**

| **Material & Methods** |  |
| --- | --- |
| ***Candidate gene selection*** |  |
| Bioinformatic pipeline | Page 2 |
| Overview of codeML’s different selection models | Page 3 |
| ***DNA extraction*** |  |
| Map of sampling locations | Page 3 |
| Overview of samples per species | Page 4 |
| ***Amplification and Sequencing of the ITS1, COI and HSP 40kDa*** |  |
| Polymerase chain reaction protocol | Page 6 |
| Thermocycler temperature program COI | Page 6 |
| Thermocycler temperature program ITS1 | Page 6 |
| Designed primer pairs HSP 40kDa | Page 6 |
| Thermocycler temperature program HSP 40kDa | Page 7 |
| Consensus sequences of the *Brachionus* species | Page 7 |
| ***Selection tests and divergence times*** |  |
| Phylogeny based on ITS1 | Page 8 |
| PAML – codeML settings | Page 9 |
| **Results** |  |
| ***HSP 40kDa Sequence Diversity*** | Page 10 |
| Structural Variation between *B. calyciflorus* s.s. and *B. fernandoi* | Page 11 |
| Congruence among ITS1, HSP 40kDa, and COI - HSP 40kDa – ITS1vs. COI | Page 12 |
| Congruence among ITS1, HSP 40kDa, and COI - ITS1 vs. HSP40kDa | Page 13 |
| Divergence time estimation | Page 14 |
| References | Page 15 |

*Methods*

*Candidate gene selection*

*
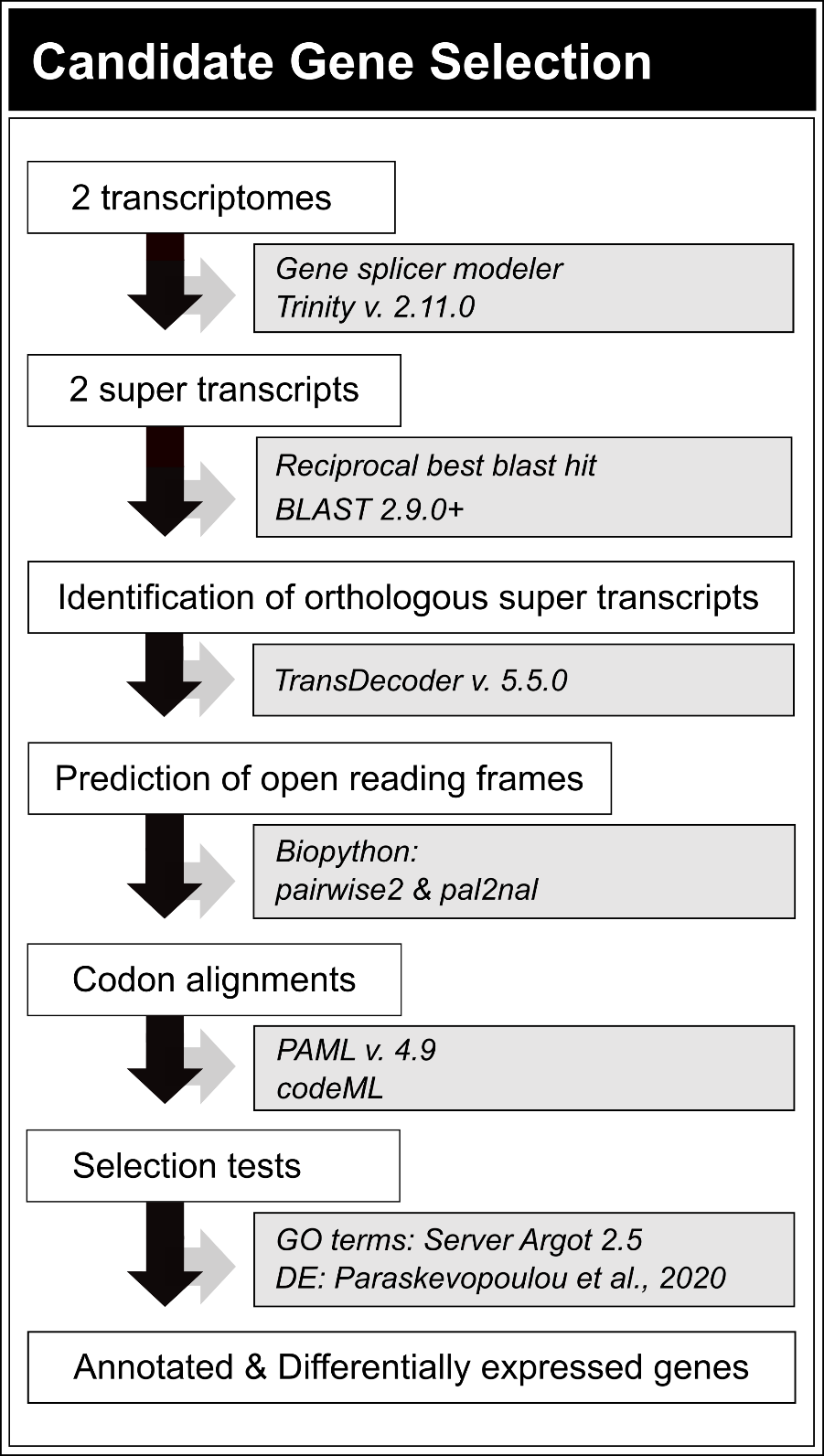
*

**Figure s1**: Bioinformatic candidate gene selection pipeline. Two published transcriptomes^[1]^ were used to generate two super transcripts using Trinity (v. 2.11.0) gene splicer modeler. Orthologous super transcripts were identified using a reciprocal best blast hit (BLAST 2.9.0+), non-coding parts were removed from the super transcripts using a TransDecoder v. 5.5.0 and open reading frames were predicted. Sequences were translated into proteins and locally aligned using Biopythons pairwise2 and pal2nal. Subsequent selection tests were conducted using codeML implemented in PAML v. 4.9. Significant orthologous genes were annotated with GO terms using the online Server Argot 2.5 and differentially expressed genes were compared using expression data^[1]^.

| **Model** | **Characteristics** |
| --- | --- |
| **M0** (one ratio) | Assumes one ω (=d_N_/d_S_) for all codon sequence |
| **M3** (discrete) | Uses an unconstrained discrete distribution with three site classes estimated for the data |
| **M1a** (nearly neutral) | Assumes two site classes estimated from the data, with ω_0_ < 1 and ω_1_ = 1 |
| **M2a** (positive selection) | Adds a third class of sites to M1a, with ω_2_ > 1 |
| **M7** (β) | Is a flexible null model, in which the ω ratio for a codon is randomly drawn from a β distribution with 0 < ω < 1 |
| **M8** (β & ω > 1) | Adds an extra class of sites to model M7, with a proportion of ω > 1 estimated from the data |
| **M8a** (β & ω = 1) | Introduced by Swanson et al. 2003 similar to M8 except that ω = 1 is fixed which does not allow positively selected sites |

**Table s1:** Different models implemented in the site model test of codeML^[2]^, with respective ω ratio of synonymous (d_S_) and non-synonymous sites (d_N_).


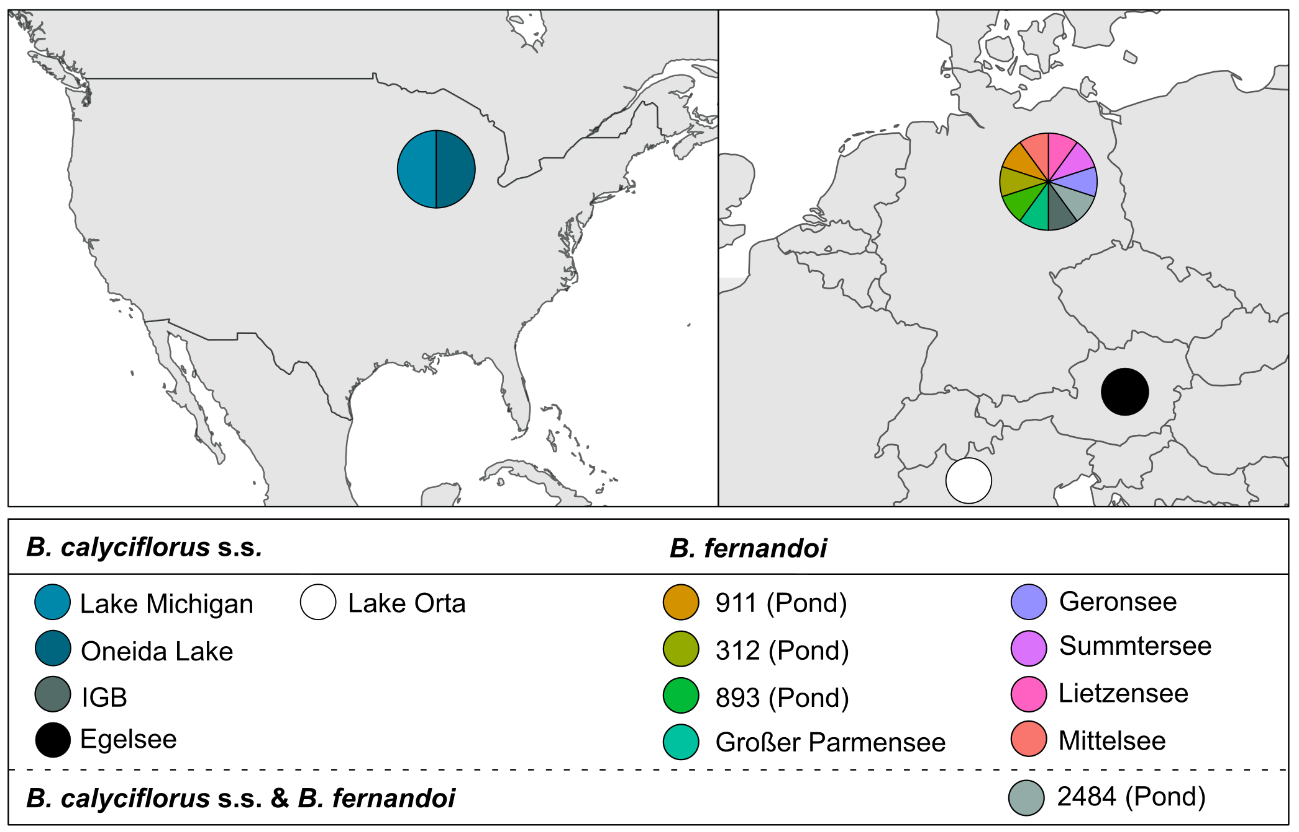
*DNA extraction*

**Figure s2**: Map of sampling locations. Origin of the samples of B. calyciflorus s.s. and B. fernandoi used in this study. The main region sampled was northeastern Germany, while some additional samples for B. calyciflorus s.s. were derived from USA, Italy, and Austria (for details see Supplementary table s2). The figure was generated with R version 4.0.5^[3]^ using the R package rnaturalearth^[4]^ and adapted in Inkscape version 1.0.1.^[5]^

**Table s2:** Overview of all samples per species, their country of origin and the analysed genetic markers as well as their HSP 40kDa alleles.

| **Species** | **Sample** | **Country of origin** | **Region** | **Location (Pond/Lake)** | **Coordinates** | **ITS** | **COI** | **HSP** | **Allele types** |
| --- | --- | --- | --- | --- | --- | --- | --- | --- | --- |
| ***B. calyciflorus* s.s.** | 893 | Germany | Uckermark | 893 | 53°24'30.08"N; 13°39'10.52"E | x | x | x | A15/A15 |
|  | 893_1 | Germany | Uckermark | 893 | 53°24'30.08"N; 13°39'10.52"E | x | x | x | A15/A15 |
|  | 893_2 | Germany | Uckermark | 893 | 53°24'30.08"N; 13°39'10.52"E | x | x | x | A15/A15 |
|  | 893_3 | Germany | Uckermark | 893 | 53°24'30.08"N; 13°39'10.52"E | x | x | x | A15/A15 |
|  | 893_5 | Germany | Uckermark | 893 | 53°24'30.08"N; 13°39'10.52"E | x | x | x | A15/A15 |
|  | 893_6 | Germany | Uckermark | 893 | 53°24'30.08"N; 13°39'10.52"E | x | x | x | A15/A15 |
|  | 893_7 | Germany | Uckermark | 893 | 53°24'30.08"N; 13°39'10.52"E | x | x | x | A15/A15 |
|  | 893_8 | Germany | Uckermark | 893 | 53°24'30.08"N; 13°39'10.52"E | x | x | x | A15/A15 |
|  | 893_9 | Germany | Uckermark | 893 | 53°24'30.08"N; 13°39'10.52"E | x | x | x | A15/A15 |
|  | 2484_may_2019 | Germany | Uckermark | 2484 | 53°21'7.93"N; 13°37'26.08"E | x | x | x | A16/A19 |
|  | 312 | Germany | Uckermark | 312 | 53°23'12.51"N; 13°41'10.54"E | x | x | x | A23/A24 |
|  | Michiganlake_9 | USA | Michigan | L. Michigan | 43°27'0.36"N; 87°13'19.27"W | x | x | x | A26/A27 |
|  | Michiganlake_14 | USA | Michigan | L. Michigan | 43°27'0.36"N; 87°13'19.27"W | x | x | x | A15/A15 |
|  | Egelsee_28 | Austria | Uterach | Egelsee | 47°49'57.1"N 13°30'15.7"E | x | x | x | A16/A16 |
|  | Egelsee_29 | Austria | Uterach | Egelsee | 47°49'57.1"N 13°30'15.7"E | x | x | x | A16/A16 |
|  | EGN25 | Austria | Uterach | Egelsee | 47°49'57.1"N 13°30'15.7"E | x | x | x | A31/A32 |
|  | EGNA5 | Austria | Uterach | Egelsee | 47°49'57.1"N 13°30'15.7"E | x | x | x | A29/A30 |
|  | Ortalake | Italy | Piemont | Lake Orta | 45°49'32.46"N; 8°23'33.56"E | x | - | x | A16/A16 |
|  | ORN1 | Italy | Piemont | Lake Orta | 45°49'32.46"N; 8°23'33.56"E | x | - | x | A16/A16 |
|  | ORN5 | Italy | Piemont | Lake Orta | 45°49'32.46"N; 8°23'33.56"E | x | x | x | A25/A28 |
|  | ORN7 | Italy | Piemont | Lake Orta | 45°49'32.46"N; 8°23'33.56"E | x | - | x | A16/A17 |
|  | ONEIDA | USA | NY | Oneida lake | 43° 9'53.05"N; 75°54'32.50"W | x | - | x | A20/A20 |
|  | ONDa | USA | NY | Oneida lake | 43° 9'53.05"N; 75°54'32.50"W | x | - | x | A20/A21 |
|  | Onogonada | USA | NY | Oneida lake | 43° 9'53.05"N; 75°54'32.50"W | x | - | x | A20/A22 |
|  | IGB | Germany | Berlin | NA | NA | x | x | x | A14/A14 |
| ***B. fernandoi*** | 2482_5_october_2019 | Germany | Uckermark | 2484 | 53°21'7.93"N; 13°37'26.08"E | x | x | x | A9/A10 |
|  | 911_3_march_2020 | Germany | Uckermark | 911 | 53°24'30.87"N; 13°38'24.48"E | x | x | x | A2/A2 |
|  | 2484_J | Germany | Uckermark | 2484 | 53°21'7.93"N; 13°37'26.08"E | x | x | x | A1/A1 |
|  | 2484_L | Germany | Uckermark | 2484 | 53°21'7.93"N; 13°37'26.08"E | x | x | x | A1/A2 |
|  | 2484 | Germany | Uckermark | 2484 | 53°21'7.93"N; 13°37'26.08"E | x | x | x | A1/A2 |
|  | 2484_1 | Germany | Uckermark | 2484 | 53°21'7.93"N; 13°37'26.08"E | x | x | x | A1/A11 |
|  | 2484_4 | Germany | Uckermark | 2484 | 53°21'7.93"N; 13°37'26.08"E | x | x | x | A18/A18 |
|  | 2484_9 | Germany | Uckermark | 2484 | 53°21'7.93"N; 13°37'26.08"E | x | x | x | A1/A1 |
|  | 2484_10 | Germany | Uckermark | 2484 | 53°21'7.93"N; 13°37'26.08"E | x | x | x | A2/A12 |
|  | 312_1 | Germany | Uckermark | 312 | 53°23'12.51"N; 13°41'10.54"E | x | x | x | A6/A7 |
|  | 312_J | Germany | Uckermark | 312 | 53°23'12.51"N; 13°41'10.54"E | x | x | x | A1/A8 |
|  | GP_5_march_2020 | Germany | Uckermark | Gr. Parmersee | 53°21'20.83"N; 13°35'30.38"E | x | x | x | A5/A5 |
|  | Summtersee_1 | Germany | Mühlenbecker Land | Summtersee | 52°41'34.69"N; 13°22'21.05"E | x | x | x | A2/A2 |
|  | Summtersee_2 | Germany | Mühlenbecker Land | Summtersee | 52°41'34.69"N; 13°22'21.05"E | x | x | x | A2/A13 |
|  | Geronsee_2 | Germany | Gransee | Geronsee | 53° 1'4.97"N; 13°10'11.54"E | x | x | x | A1/A4 |
|  | Lietzensee_A1 | Germany | Berlin | Lietzensee | 52°30'27.45"N; 13°17'16.75"E | x | x | x | A1/A1 |
|  | Lietzensee_A10 | Germany | Berlin | Lietzensee | 52°30'27.45"N; 13°17'16.75"E | x | x | x | A1/A1 |
|  | Mittelsee_1 | Germany | Höhenland | Mittelsee | 52°41'06.3"N 13°51'37.4"E | x | x | x | A3/A3 |
|  | Mittelsee_2 | Germany | Höhenland | Mittelsee | 52°41'06.3"N 13°51'37.4"E | x | x | x | A3/A3 |
| ***B. rubens*** | 258_8_april_2019 | Germany | Uckermark | 258 | 53°22'56.84"N; 13°42'25.11"E | x | x | x | Consensus sequence |
|  | 258_2_april_2019 | Germany | Uckermark | 258 | 53°22'56.84"N; 13°42'25.11"E | x | x | x |  |
|  | 18_1_june_2019 | Germany | Uckermark | 18 | 53°19'24.97"N; 13°31'44.76"E | x | - | x |  |
|  | 18_1_may_2020 | Germany | Uckermark | 18 | 53°19'24.97"N; 13°31'44.76"E | x | x | x |  |
| ***B. angularis*** | 2484_3_june_2019 | Germany | Uckermark | 2484 | 53°21'7.93"N; 13°37'26.08"E | x | x | x | Consensus sequence |
|  | 2484_4_june_2019 | Germany | Uckermark | 2484 | 53°21'7.93"N; 13°37'26.08"E | x | x | x |  |
|  | 2484_5_june_2019 | Germany | Uckermark | 2484 | 53°21'7.93"N; 13°37'26.08"E | x | - | x |  |
|  | 2484_8_june_2019 | Germany | Uckermark | 2484 | 53°21'7.93"N; 13°37'26.08"E | x | - | x |  |
|  | 2484_10_june_2019 | Germany | Uckermark | 2484 | 53°21'7.93"N; 13°37'26.08"E | x | x | x |  |
| ***B. diversicornis*** | 2484_1_march_2019 | Germany | Uckermark | 2484 | 53°21'7.93"N; 13°37'26.08"E | x | - | x | Consensus sequence |
|  | 911_17_march_2020 | Germany | Uckermark | 911 | 53°24'30.87"N; 13°38'24.48"E | x | x | x |  |
|  | GP5_may_2019 | Germany | Uckermark | Gr. Parmersee | 53°21'20.83"N; 13°35'30.38"E | x | x | x |  |

*Amplification and Sequencing of the ITS1, COI and HSP 40kDa*

| **Chemical** | **Volume [µl]** |
| --- | --- |
| 5x My-Taq buffer | 5.0 |
| Forward-Primer [10µM] | 0.5 |
| Reverse-Primer [10µM] | 0.5 |
| My-Taq | 0.12 |
| HPLC-H_2_O | 15.88-17.88* |
| DNA | 1-3* |

**Table s3**: Polymerase chain reaction protocol. Volumes marked with an asterisk were adjusted according to the DNA volume used.

**Table s4:** Thermocycler temperature program for the COI Polymerase chain reaction, using the universal invertebrate primers^[6]^. Step 1-4 was performed in 40 cycles.

| **Reaction step** | **Temperature [°C]** | **Time** | **Primer sequences** |
| --- | --- | --- | --- |
| 1. Initial denaturation | 96.0 | 5 min |  |
| 2. Denaturation | 96.0 | 15 sec |  |
| 3. Primer annealing | 52.0 | 1 min | LCO1490: GTCAACAAATCATAAAGATATTGG  HCO2198: TAAACTTCAGGGTGACCAAAAAATCA |
| 4. Elongation | 72.0 | 45 sec |  |
| 5. Final Elongation | 72.0 | 10 min |  |
| 6. Pause | 10.0 | ∞ |  |

**Table s5:** Thermocycler temperature program for the ITS Polymerase chain reaction, using the universal invertebrate primers^[7]^. Step 1-4 was performed in 40 cycles.

| **Reaction step** | **Temperature [°C]** | **Time** | **Primer sequences** |
| --- | --- | --- | --- |
| 1. Initial denaturation | 96.0 | 5 min |  |
| 2. Denaturation | 96.0 | 15 sec |  |
| 3. Primer annealing | 55.0 | 1 min | VIII: GTGCGTTCGAAGTGTCGATGATCAA  III: CACACCGCCCGTCGCTACTACCGATTG |
| 4. Elongation | 72.0 | 45 sec |  |
| 5. Final Elongation | 72.0 | 10 min |  |
| 6. Pause | 10.0 | ∞ |  |

**Table s6:** Primer pairs used in the study to amplify HSP 40kDa. Name, length, primer sequence, expected product size and annealing temperature used are given. Two different primer pairs were used to amplify the HSP 40kDa from B. rubens, B. angularis and B. diversicornis and sequences were subsequently aligned to generate the full sequence.

| **Species** | **Name** | **Length [bp]** | **Primer sequence 5’ – 3’** | **Product size**  **[bp]** | **Annealing temperature** |
| --- | --- | --- | --- | --- | --- |
| ***B. calyciflorus* s.s.** | B.caly_HSP40_F | 20 | CAAACCAAGTGCTTCAGCTG | 1167 | 57°C |
|  | B.caly_HSP40_R | 19 | TTGGCACATTGGACTCGTT |  |  |
| ***B. fernandoi*** | B.fern_HSP40_B.caly_F | 20 | CAAACCAAGCGCCACTCCTG | 1167 | 57°C |
|  | B.caly_HSP40_R | 19 | TTGGCACATTGGACTCGTT |  |  |
| ***B. rubens***  ***B. angularis***  ***B. diversicornis*** | Brach-HSP40_F | 19 | GMAAMATGGTKAAAGAAAC | 669 | 40°C |
|  | Brach-HSP40_Rint | 25 | GCATACCYTTRTCAATRTGAACYTC |  |  |
|  | Brach-HSP40_Fint | 24 | GTCTAAGCHTAYGAAGTATTGAG | 1055 | 43°C |
|  | B.caly_HSP40_R | 19 | TTGGCACATTGGACTCGTT |  |  |

**Table s7:** Thermocycler temperature program for the HSP 40kDa polymerase chain reaction. The asterisk is a placeholder for the different annealing temperatures given in table s6. Step 1-4 was performed in 40 cycles.

| **Reaction step** | **Temperature [°C]** | **Time** |
| --- | --- | --- |
| 1. Initial denaturation | 95.0 | 5 min |
| 2. Denaturation | 95.0 | 10 sec |
| 3. Primer annealing | * | 40 sec |
| 4. Elongation | 72.0 | 1 min |
| 5. Final Elongation | 72.0 | 10 min |
| 6. Pause | 10.0 | ∞ |

**Table s8:** 1,025 bp long consensus sequences of the HSP 40kDa which was used to assess the sequence diversity within the Brachionus calyciflorus species complex (i.e., B. calyciflorus s.s. and B. fernandoi) and across different species (i.e., B. rubens, B. angularis, B. diversicornis). For underlying clone information see supplemental table s2.

| **Species** | **Sequence 5’ – 3’** |
| --- | --- |
| *B. calyciflorus* s.s. | ATTGAAAAAAGCCTATCGNAAATTGGCGCTTTAATACCATCCGGANAAGAATCCAGACAAAGATTCGGCCGAAAAATTCAAAAAAACTCTCAAGCTTATGAAGTATTGAGTGATGAGAAAAAAAGACACATCTATGATGAAGGTGGTGAACAAGCGCTTAAGGAAGGCGGTGGTGGCGAGGGTCATTTCAGTTCACCAATGGATATTTTCGAGATGTTCTTCGGCGGTGGTGGTGGCGGTCGTAGAAGAAAAGAAAACAAAGGAAAAGATGTAATACATCAATTGGGGGTTACTTTAGAAGATTTGTACAAGGGTTCAACTAGGAAATTAGCTTTGCAAAAAAATGTTATTTGTGATAAATGTAGTGGAAAGGGTGGTAAAGAGGGTGCTGTGATTAGATGTACAACATGTAAGGGTAGTGGTACACAAGTTATTTTAAATCAATTAGGAGCTGGTATGTACCAACAAATACATACTTCTTGTCGCGATTGTGGTGGTCAAGGTGAAAAAATAAATCCAAAAGATATGTGTAAAACTTGTCAAGGCAAAAAAATTGTACAAGAAAGAAAAATATTGGAAGTTCATATCGATAAAGGTATGGAAGACGGACAAAAAATATTTTTCTACGGTGAAGGTGATCAATCACCAGGTCTAGAACCAGGAGATATCATAATTATTTTAGAAGAGAAAGAGCATTCTGTTTTCAGACGTAAAGATATGGATTTATTAATGAAAATGGAAATTAATTTGAATGAGGCTTTGACTGGTTTTAGAAGAACTATTAAAACTTTGGACGATAGAATTTTAGTTATTTCTAGTCACCCAGGCGAGTTTATAAAACCCAATGACATTAAATGTGTTTTGAATGAGGGTATGCCAATGTACAAAAATCCATTCGAAAAAGGTCGTTTGATAATAACATTTAGTGTTAAATTTCCTCAAAATGGAGACATTGAATTAAAAAAAATCACTGAATTGGAAAAAATATTGCCTGCAAAACAAAAAGCAGATGCACCTGC |
| *B. fernandoi* | ATTGNAGAACGCCGACCGCAGGTTCGGATTGNAAATATCATCCGGANAAAAACCCCGACCAAGATTCGGCCGAAAAATTCAAAAAAACTCACAAGCCTATGAAGTATTGAGCGACGAGAAAAAAAGAGTAATCTATGATGAAGGCGGTGAACAAGCGCTTAAGGAAGGCGGTGGTGGCGGGGGTCATTCGCCAATGGATATTTTCGAGATGTTCTTCGGCGGCGGCGGTGGTGGTCGTAGAAGAAAAGAGAATAAAGGCAAAGATGTCATACATCAAATGGGGGTTTCTTTAGAAGATTTGTATAAAGGTTCAACTAGGAAATTAGCTTTACAAAAGAATGTTATTTGTGATAAATGCAGCGGAAAAGGTGGAAAAGAGGGCGCTGTTATACGATGTACTACTTGTAAGGGGTCTGGTACACAGGTTATTTTAAACCAGTTAGGAGCGGGTATGTATCAACAAATACATGCTTCTTGTCGAGATTGTGGCGGACAGGGTGAAAAAATAAATCCAAAAGACATGTGTAAAACTTGTCAAGGCAAAAAAATTGTTCAGGAAAGAAAAATCTTAGAAGTTCATATTGACAAAGGTATGGAAGACGGGCAAAAAATCGTTTTCAATGGTGAAGGTGATCAATCGCCAGGCCTAGAACCAGGAGATATCATAATTATCCTTGAAGAAAAAGAACATTCGGTCTTTAGACGTAAAGATATGGATTTACTTATGAAAATGGAAATAAATTTGAATGAGGCGTTGACTGGTTTTAAGCGAACTATTAAAACTTTGGACGATAGAATTCTAGTTATTTCTAGTCTACCGGGTGAATTTATAAAGCCTAATGATATTAAATGTGTTTTGAATGAAGGTATGCCTATGTATAAAAATCCGTTCGAAAAAGGTCGTTTAATAATAACATTCAGTGTTAAATTTCCTCAAAATGGAGAGATAGAATTGAAAAAAATTCGCGAATTGGAAAAAATATTGCCACTTAAGCAAAAGGCAGATGCGCCGGT |
| *B. angularis* | GCTCAAAAAAGCCTATCNGTAAACTTGCGCTTAAGTTTCATCCAGACAAAAATCCAGACAAAGATTCGGCCGAAAAATTTAAAAATATCTCTCAAGCTTACGAAGTACTGAGTGATGAGAAAAAAAGACGTATTTATGATGAAGGCGGTGAGCAAGCGTTGAAAGAAGGTGGAACTGGAGAAGGTCATTTTAGCTCGCCAATGGACATTTTTGAAATGTTTTTCGGTGGCGGAGGTGGTGGTCGCAGACGTAAAGAAAATAAAGGCAAAGATGTTATTCATCAAATGGGAGTTTCTTTAGAAGATTTGTACAAAGGTTCAACTAGGAAATTAGCTCTTCAAAAAAATGTTATCTGTGATAAATGTGCAGGTAAAGGAGGCAAAGAAGGTGCTGTTATCACATGTACAACATGTAAGGGCAGTGGTAGTCAAGTCATATTGAATCAATTAGGNGCTGGAATGTATCAACAAATACATACAAGTTGTAGAGATTGTGGTGGTCAAGGAGAAAAAATAAATCCAAAAGACATGTGTAAAACTTGTCAGGGTCGCAAGATCGTCCAAGAACGAAAAATATTGGAAGTCCACATCGACAAAGGTATGGAAGATGGACAAAAAATTGTATTCTATGGAGAAGGAGACCAATCGCCAGGTCTTGAACCAGGAGATATAATTATTATATTAGAAGAAAAAGAACATGCAACATATAAACGTAAAGACATGGACCTTTATATGAAAATGGACATTAATTTGAATGAAGCTTTGACTGGTTTTAAACGAACAATTAAAACTTTGGATGATAGAATTTTGGTAATTAATAGTCATCCAGGAGAAATAATTAAACAAAACGCAGTTAAATGTGTATTGAATGAGGGAATGCCAATGTATAAAAACCCGTTCGAAAAAGGTCGTTTGATAATCACTTTCAATGTAAAATTTCCACAAAATGGAGAAATTGAGTTGAAAAAGATTAGTGAATTGGAAAAAATATTGCCAGCAAAACCGAAAATAGAAGCACCAGC |
| *B. rubens* | GCTTAAAAAAGCATATCGAAAATTGGCTCTTAAATTCCATCCAGACAAAAATCCAGACAAAGATTCGGCCGAAAAATTCAAAAATATATCACAAGCATATGAGGTATTAAGTGATGAGAAAAAGAGACATATTTATGATGAAGGCGGTGAACAAGCATTGAAAGAAGGTGGTGGCGGCGAAGGCCATTTCAGTTCGCCAATGGACATTTTTGAAATGTTCTTTGGTGGCGGTGGTGGAGGCCGACGACGAAAAGAAAACAAAGGAAAAGACGTCATACACCAATTAAGTGTTACACTTGAAGATCTATACAAAGGGTCAACTAGAAAATTAGCTTTACAAAAGAATGTCATTTGTGATAAATGTGCTGGTAAAGGAGGTAAAGAAGGCGCCGTTCTCAAGTGTACAACTTGTAAAGGAAGCGGTAGTCAAGTTATTTTGAACCAATTAGGTGCTGGTATGTACCAACAAATCCACACTACATGCAGAGAGTGTCAAGGTCAGGGTGAAAAAATAAATCCAAAAGATATGTGTAAAACTTGTCAAGGTCGTAAAATTGTTCAAGAAAGAAAAATCTTAGAAGTTCACATTGACAAGGGTATGGAAGATGGGCAAAAAATTGTTTTCTATGGTGAGGGTGATCAATCACCAGGTCTAGAACCAGGTGATATAATCATCATATTGGAAGAAAAAGAACATTCTTTATTTAAACGAAGAGACATGGATCTTAACATGAAAATGGAGATTAATTTGAACGAAGCACTTACTGGTTTTAGGCGCACGATTAAAACTCTTGATGACAGAATTTTGGTTATTTCTACTATACCAGGCGATTTCGTAAAACCAAACGAAGTTAAATGTGTTTTGAACGAAGGTATGCCAATGTATAAAAATCCGTTCGAAAAAGGTCGTTTGATAATTACATTTAATGTAAAATTTCCTCAAAATGGTGAAATTGAAATGAAAAAAATAGCCGATTTGGAAAAAATATTGCCAGCAAAAGCAAAAGTTGAAGCCCCCGC |
| *B. diversicornis* | ACTAAAAAAAGCCTATCGAAAACTTGCGCTCAAGTTTCATCCAGACAAAAATCCAGACAAAGACTCGGCCGAAAAATTCAAAAACATCTCTCAAGCATACGAAGTATTGAGTGATGAGAAAAAGAGACGATCTTATGACGAAGGAGGCGAACAAGCGCTAAAAGAAGGTGGAAGTGGAGAAGGTCATTTCAGTTCGCCAATGGACATTTTCGAAATGTTTTTCGGTGGCGGTGGTGGTGCCGGTCGTAGACGTAAAGAAAATAAAGGCAAAGATGTTATTCATCAATTGGGAGTTACTTTAGAAGATTTGTACAAAGGCTCAACTAGGAAATTGGCTCTTCAAAAAAATGTAATTTGTGATAAATGTAATGGTAAAGGAGGTAAAGAAGGTGCTGTTATTTCATGCACAACATGTAAGGGTAGTGGTAGTCAAGTTATATTGAATCAATTAGGCGCTGGTATGTACCAACAAATACACACATCATGTAGAGATTGTGGTGGTCAAGGTGAAAAAATTAATCCCAAAGACATGTGCAAGACTTGTCAAGGGCGCAAAATCGTTCAAGAAAGAAAAATATTAGAGGTTCACATTGACAAAGGTATGGAAGACGGTCAAAAAATTTTTTTCTATGGAGAAGGTGATCAATCACCAGGTCTTGAACCAGGAGATATCATTATTATCTTAGAAGAAAAAGAACATACACTATTTAGACGTAAAGACATGGATCTATATATGAAAATGGAGATTAATTTGAATGAGGCTTTGACTGGTTTTAGACGTACAATTAAAACTTTAGATGATAGAATTTTGGTAATTTCTAGTCATCCAGGAGAATTTATTAAACAAAATGATGTAAAATGTGTTTTAAATGAGGGTATGCCAATGTATAAAAACCCGTTCGAAAAAGGTCGTTTAATAATCACTTTCAATGTTAAATTTCCAAAAAATGGCGACATAGAATTGAAAAAAATTGTCGAATTGGAAAAAATATTACCAGCAAAACCGAAAATAGAAGCACCGGC |

*Selection tests and divergence time*


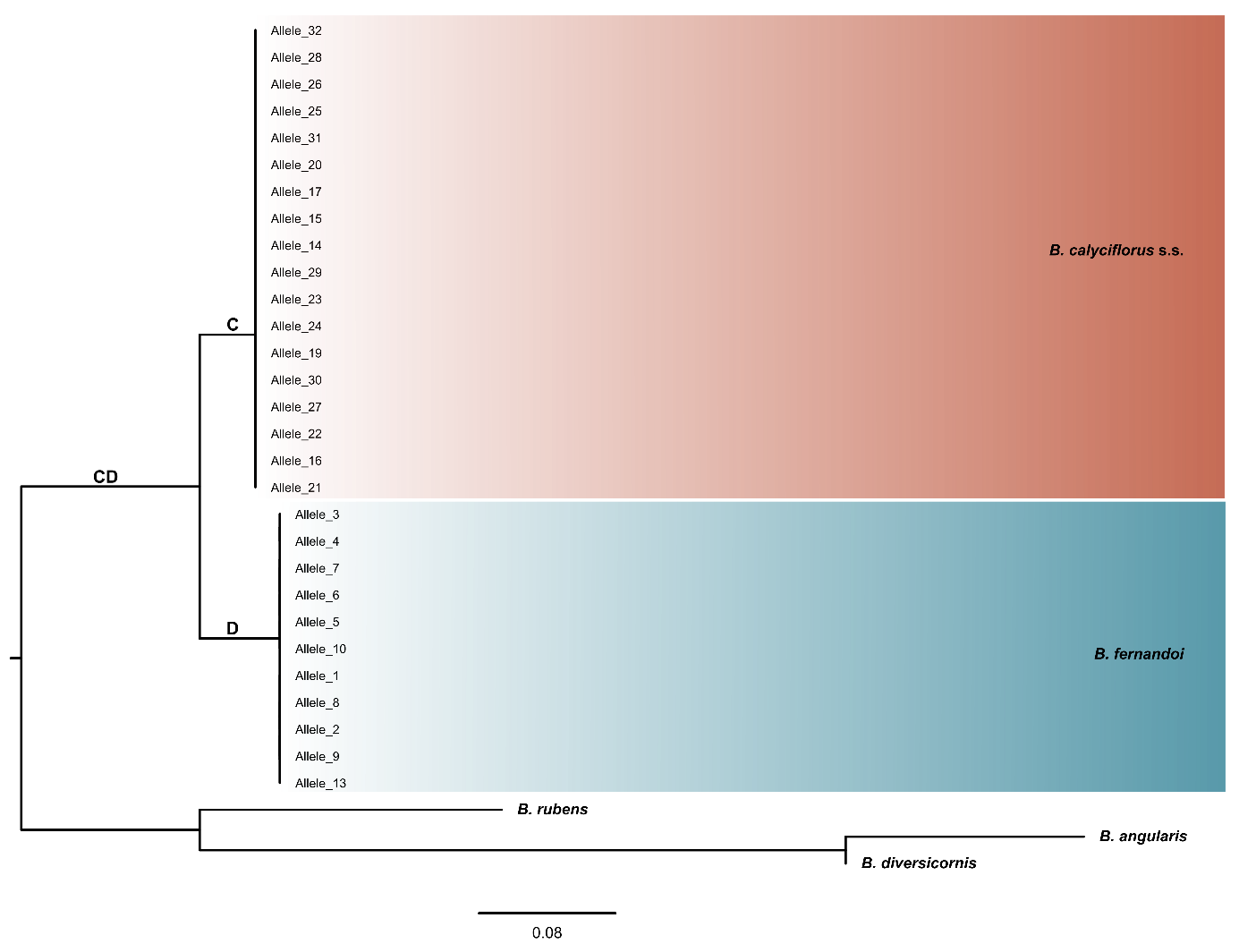


**Figure s3**: ITS1 Phylogeny of different Brachionus species. Phylogenetic tree used for the selection test of codeML site model and branch site model, based on 532 bp long ITS1 sequence alignment corresponding to the 29 unique HSP 40kDa alleles. Three alleles (A11, A12 and A18) originated from hybrids between B. calyciflorus s.s. and B. fernandoi and were therefore excluded from the analysis. The branch labels CD, C and D correspond to the branches selected in the branch site model implemented in codeML.

**Table s9:** Settings used in PAML - codeML site model to test for positively selected sites in the HSP 40kDa.

| **codeML site model settings M0-M8** |
| --- |
| noisy = 4 |
| verbose = 1 |
| runmode = 0 |
| seqtype = 1 |
| CodonFreq = 2 |
| estFreq = 0 |
| ndata = 32 |
| clock = 0 |
| aaDist = 0 |
| model = 0 |
| NSsites = 0 1 2 3 7 8 |
| icode = 0 |
| Mgene = 0 |
| fix_kappa = 0 |
| kappa = 2 |
| fix_omega = 1 |
| omega = 0.4 |
| fix_alpha = 1 |
| alpha = 0 |
| Malpha = 0 |
| ncatG = 5 |
| getSE = 1 |
| RateAncestor = 0 |
| Small_Diff = 5e-7 |
| cleandata = 0 |
| fix_blength = 0 |
| method = 0 |
| **codeML site model settings M8a** |
| noisy = 4 |
| verbose = 1 |
| runmode = 0 |
| seqtype = 1 |
| CodonFreq = 2 |
| estFreq = 0 |
| ndata = 32 |
| clock = 0 |
| aaDist = 0 |
| model = 0 |
| NSsites = 8 |
| icode = 0 |
| Mgene = 0 |
| fix_kappa = 0 |
| kappa = 2 |
| fix_omega = 1 |
| omega = 0.4 |
| fix_alpha = 1 |
| alpha = 0 |
| Malpha = 0 |
| ncatG = 5 |
| getSE = 1 |
| RateAncestor = 0 |
| Small_Diff = 5e-7 |
| cleandata = 0 |
| fix_blength = 0 |
| method = 0 |

*Results*

*HSP 40kDa Sequence Diversity*

*
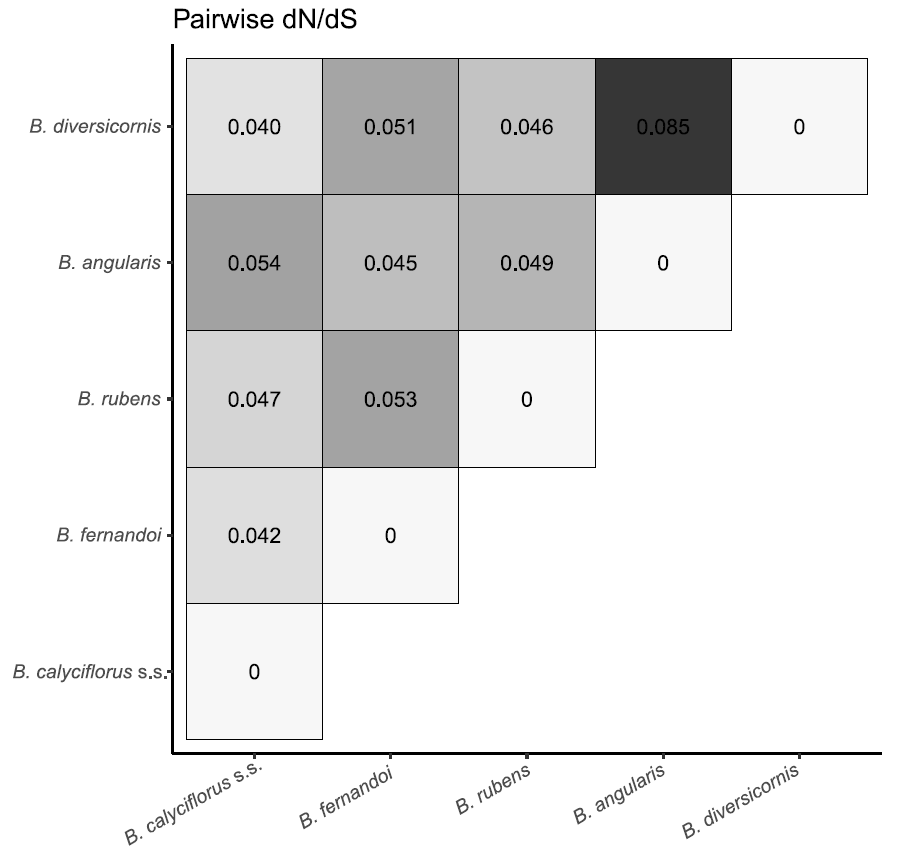
*

**Figure s4**: Synonymous and non-synonymous substitutions of the HSP 40kDa. Pairwise comparison of non-synonymous and synonymous substitutions of the HSP 40kDa gene among different Brachionus species (B. calyciflorus s.s., B. fernandoi, B. rubens, B. angularis, and B. diversicornis). The colour intensity indicates increasing dN/dS differences between the compared sequences.


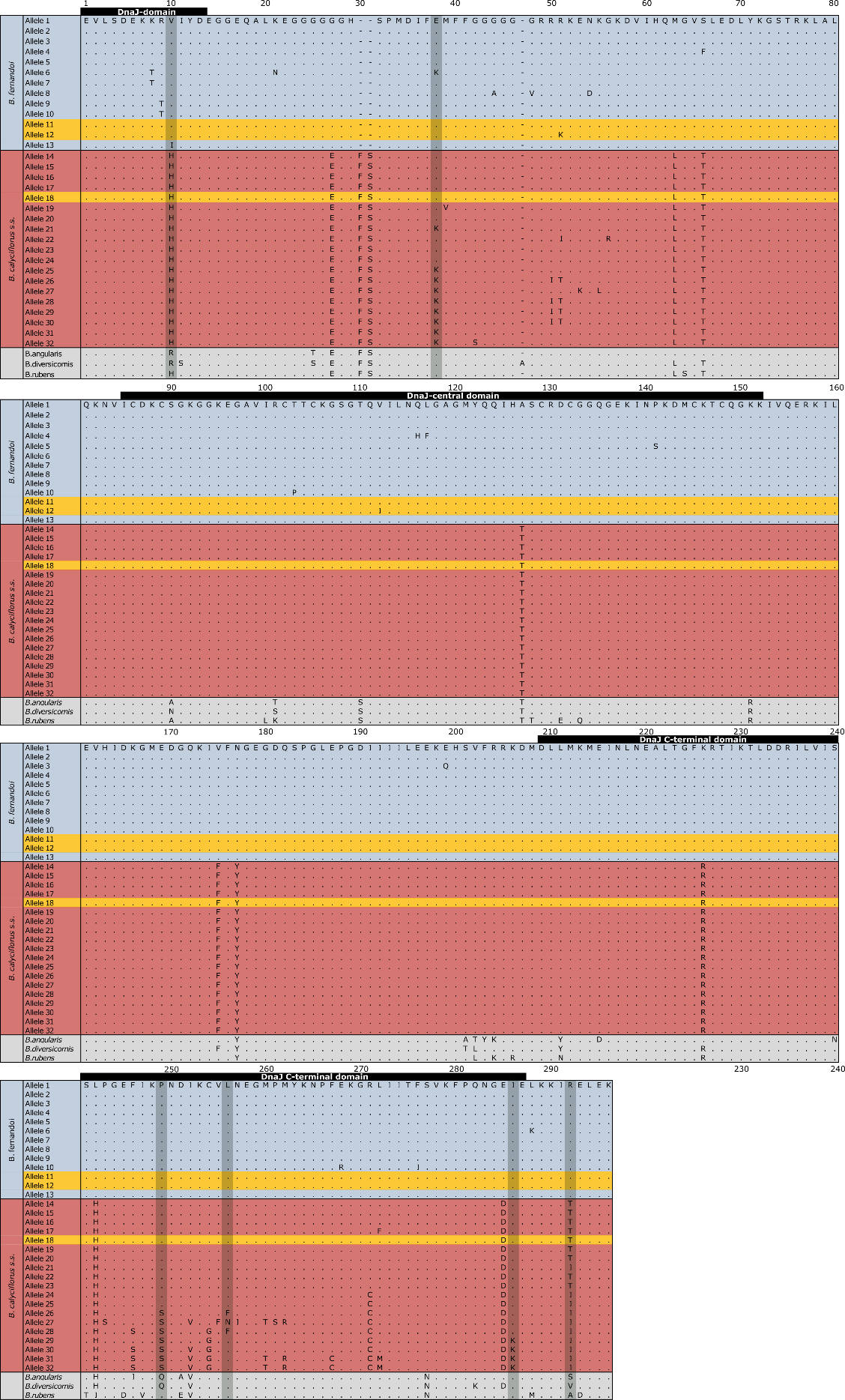
*Structural Variation between B. calyciflorus* s.s. *and B. fernandoi*

**Figure s5**: Analysed 296 amino acids of the HSP 40kDa. Alignment of the 32 unique alleles discovered in B. calyciflorus s.s. (allele 14-32, red), B. fernandoi (allele 1-13, blue). B. angularis, B. diversicornis, and B. rubens were used as outgroups. Alleles derived from descendants of hybrids are coloured in yellow. Sites under positive selection (cf. table s2) are highlighted in grey. Functional description, indicated with black bars derived from the published B. calyciflorus s.s. HSP 40kDa sequence^[8]^. Alignment was adapted using Inkscape version 1.0.1.

*Congruence among ITS1, HSP 40kDa, and COI species affinity*


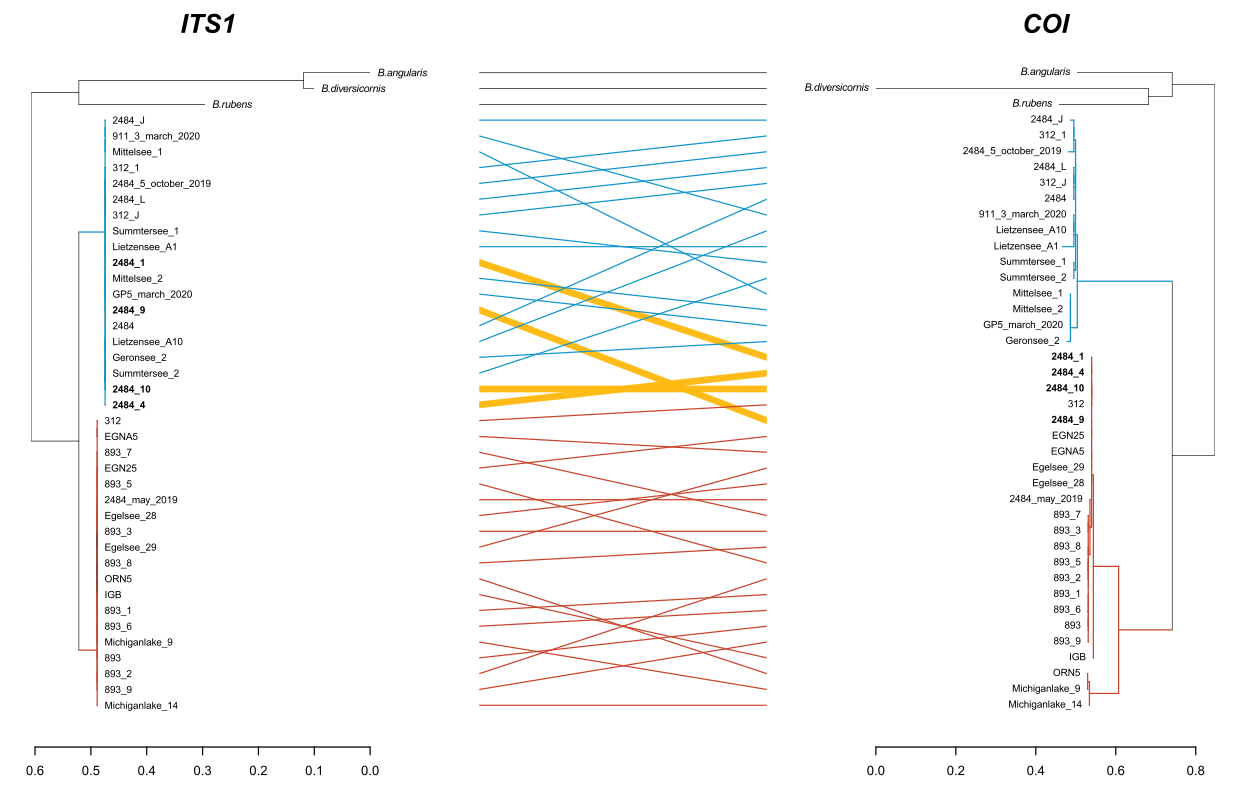


**Figure s6**: Relationship between and ITS1 and COI (n = 41). Tanglegram based on the nuclear HSP 40kDa and the ITS1 sequence of species B. calyciflorus s.s., B. fernandoi, B. rubens, B. angularis, and B. diversicornis. Black lines indicate connections of specimens outside of the Brachionus calyciflorus species complex, blue lines indicate connections between specimens of B. fernandoi and red lines connect specimens of B. calyciflorus s.s. Yellow coloured connections indicate recent introgression/hybridization events between B. calyciflorus s.s. and B. fernandoi.


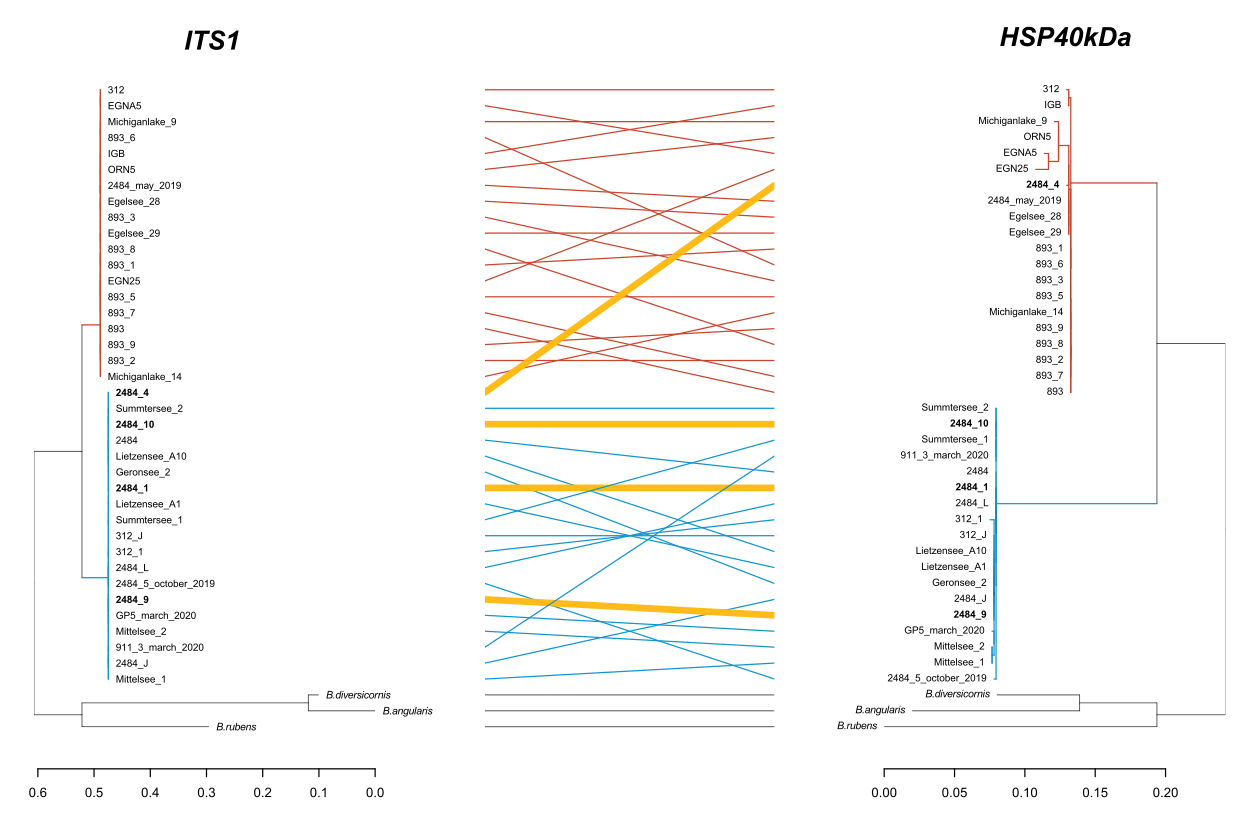


**Figure s7**: Relationship between ITS1 and HSP 40kDa (n = 41). Tanglegram based on the nuclear ITS1 and the mitochondrial COI sequence of species (B. calyciflorus s.s., B. fernandoi, B. rubens, B. angularis, B. diversicornis). Black lines indicate connections of specimens outside of the Brachionus calyciflorus species complex, blue lines indicate connections between specimens of B. fernandoi and red lines connect specimens of B. calyciflorus s.s. Yellow coloured connections indicate recent introgression/hybridization events between B. calyciflorus s.s. and B. fernandoi.

*Divergence time estimation*


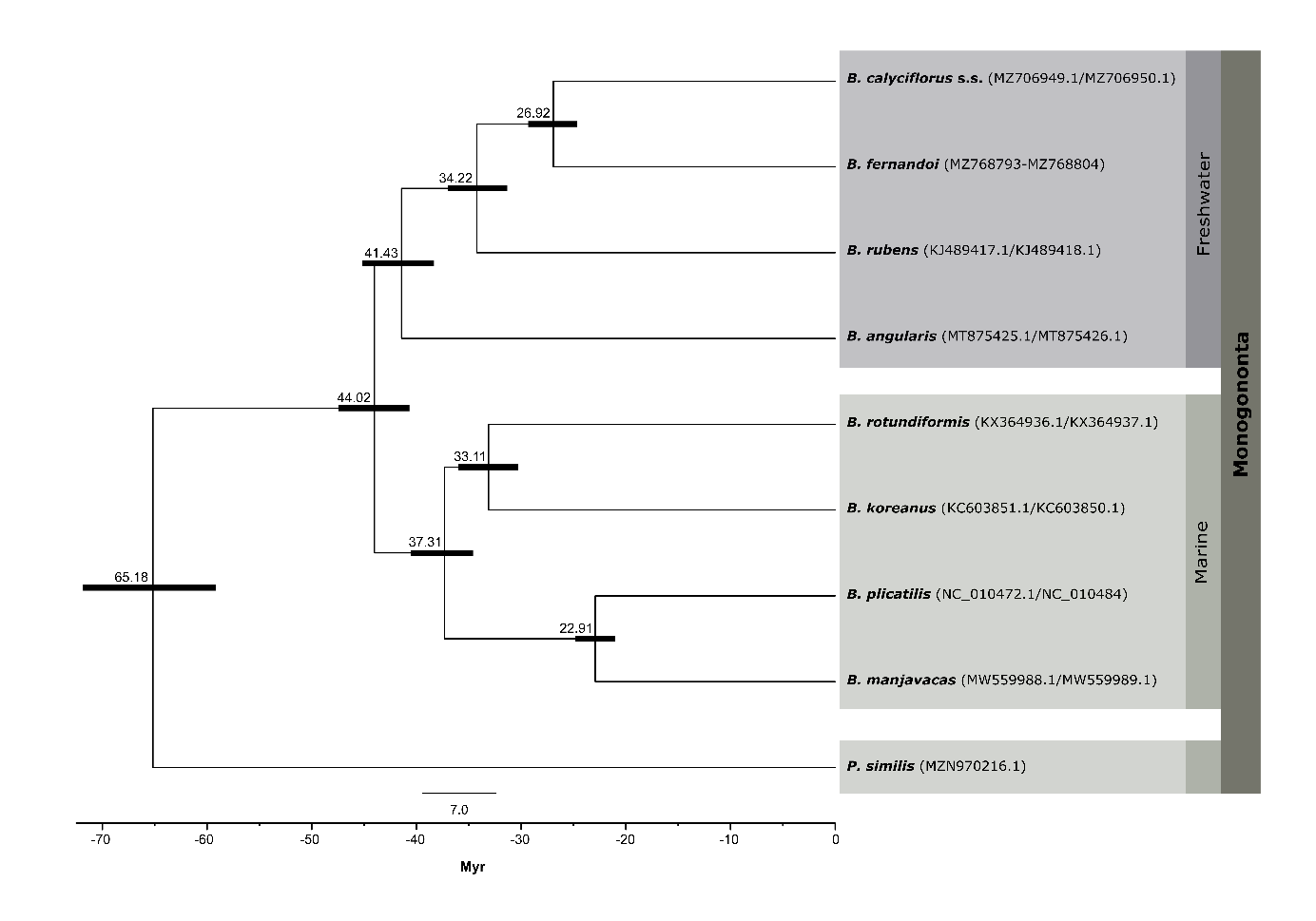


**Figure s8**: Phylogenetic trees based on cds of the mitochondrial genomes of nine different rotifer species. Trees were calibrated using the published general mitochondrial divergence rate of 2.3% Myr (substitution rate per lineages 0.0115 per million years^[9]^). The labels on the nodes indicate the estimated divergence times, the node bars show the 95% confidence interval of calculated divergence times.

*References*

[1] Paraskevopoulou, S., Dennis, A. B., Weithoff, G. & Tiedemann, R. Temperature-dependent life history and transcriptomic responses in heat-tolerant versus heat-sensitive *Brachionus* rotifers. *Sci. Rep.* **10**, 13281 (2020).

[2] Nielsen, R. & Yang, Z. Likelihood models for detecting positively selected amino acid sites and applications to the HIV-1 envelope gene. *Genetics* **148**, 929–936 (1998).

[3] Team, R. Core. R: A language and environment for statistical computing. R Foundation for Statistical Computing, Vienna, Austria. https://www.R-project.org/ (2021).

[4] South, A. Rnaturalearth: world map data from natural earth. R package version 0.1. 0, 79-88 (2017)

[5] Inkscape Project. (2020). Inkscape. Retrieved from https://inkscape.org

[6] Folmer, O., Black, M., Hoeh, W., Lutz, R., Vrijenhoek, R. DNA primers for amplification of mitochondrial cytochrome c oxidase subunit I from diverse metazoan invertebrates. *Mol. Mar. Biol. Biotechnol.* **3,** 294–299 (1994).

[7] Palumbi S.R. The polymerase chain reaction. *Mol. Syst.* **2**, 205–247 (1996).

[8] Yang, J., Mu, Y., Dong, S., Jiang, Q. & Yang, J. Changes in the expression of four heat shock proteins during the aging process in *Brachionus calyciflorus* (Rotifera). *Cell Stress Chaperones* **19**, 33–52 (2014).

[9] Brower, A. V. Rapid morphological radiation and convergence among races of the butterfly *Heliconius erato* inferred from patterns of mitochondrial DNA evolution. *Proc. Natl. Acad. Sci. U.S.A.* **91**, 6491–6495 (1994).
